# A qPCR expression assay of *IFI44L* gene differentiates viral from bacterial infections in febrile children

**DOI:** 10.1038/s41598-019-48162-9

**Published:** 2019-08-13

**Authors:** Alberto Gómez-Carballa, Miriam Cebey-López, Jacobo Pardo-Seco, Ruth Barral-Arca, Irene Rivero-Calle, Sara Pischedda, María José Currás-Tuala, José Gómez-Rial, Francisco Barros, Federico Martinón-Torres, Antonio Salas

**Affiliations:** 1 Genetics, Vaccines and Infections Research Group (GENVIP), Instituto de Investigación Sanitaria de Santiago, Santiago de Compostela, Spain; 2 Translational Pediatrics and Infectious Diseases, Department of Pediatrics, Hospital Clínico Universitario de Santiago de Compostela, Santiago de Compostela, Spain; 3 Unidade de Xenética, Instituto de Ciencias Forenses, Facultade de Medicina, Universidade de Santiago de Compostela, and GenPoB Research Group, Instituto de Investigaciones Sanitarias (IDIS), Hospital Clínico Universitario de Santiago (SERGAS), Galicia, Spain; 4 Unidad de Medicina Molecular, Fundación Pública Galega de Medicina Xenómica, CIBERER, Santiago de Compostela, Spain

**Keywords:** Transcriptomics, Bacterial infection, Gene expression

## Abstract

The diagnosis of bacterial infections in hospital settings is currently performed using bacterial culture from sterile site, but they are lengthy and limited. Transcriptomic biomarkers are becoming promising tools for diagnosis with potential applicability in clinical settings. We evaluated a RT-qPCR assay for a 2-transcript host expression signature (*FAM89A* and *IFI44L* genes) inferred from microarray data that allow to differentiate between viral and bacterial infection in febrile children. This assay was able to discriminate viral from bacterial infections (*P*-value = 1.04 × 10^−4^; AUC = 92.2%; sensitivity = 90.9%; specificity = 85.7%) and showed very high reproducibility regardless of the reference gene(s) used to normalize the data. Unexpectedly, the monogenic *IFI44L* expression signature yielded better results than those obtained from the 2-transcript test (*P*-value = 3.59 × 10^−5^; AUC = 94.1%; sensitivity = 90.9%; specificity = 92.8%). We validated this *IFI44L* signature in previously published microarray and whole-transcriptome data from patients affected by different types of viral and bacterial infections, confirming that this gene alone differentiates between both groups, thus saving time, effort, and costs. Herein, we demonstrate that host expression microarray data can be successfully translated into a fast, highly accurate and relatively inexpensive *in vitro* assay that could be implemented in the clinical routine.

## Introduction

A common practice in hospitals is to systematically administer antibiotics to febrile patients until the results from culture tests are available as preventive method, with the outcome that many viral infections are inadequately treated with anti-microbial drugs^1,2^. Moreover, it has been shown that excessive antibiotic administration may have led to an increase in bacterial resistance, not only at individual but also at more global levels^3^. Hence, misuse and overuse of the currently available therapeutic drugs is actively contributing to generate anti-microbial resistance^4–6^. This fact, together with the lack of new generation antibiotics, is making a “post-antibiotic era” a very real prospect in the near future; this is increasingly becoming a global health concern, as recently highlighted by the World Health Organization (WHO)^7^.

An early distinction between viral and bacterial patients might allow a more accurate diagnosis and treatment of the patient, reducing significantly the unnecessary use of antimicrobial drugs. In this context, the host transcriptome during the infection is proving to be a promising target to find out infectious disease-associated biomarkers^8–12^. Many studies have focused on the identification of host-specific transcriptomic biomarkers in viral and bacterial infections in both adults and children^12–18^. Out of all of these transcriptomic signatures, one in particular stands out owing to the very few mRNAs required^16^. By screening samples from febrile children using expression microarrays, Herberg *et al*.^16^ found two genes with different expression levels depending on the nature of the causative pathogen: Interferon Induced Protein 44 Like (*IFI44L*) gene is up-regulated in viral febrile children and, in contrast, Family with Sequence Similarity 89 Member A (*FAM89A*) gene has elevated expression in febrile children with bacterial infection. These biomarkers alone are capable of differentiating between bacterial and viral infections with high sensibility and specificity. These finding were subsequently replicated by Kaforou *et al*.^15^ in a cohort of febrile children under 60 days of age using the microarrays data from Mahajan *et al*.^14^. Most recently, Barral-Arca *et al*.^19^ validated this signature using whole transcriptome data from patients suffering from acute diarrhea with bacterial and viral etiology; they found that this signature clearly discriminated between viral and bacterial infections regardless of the pathogen, severity and ancestry of patients.

Even though expression microarrays are one of the most powerful screening approaches for the identification of disease-related biomarkers, findings need to be validated with highly sensitive techniques that allow the precise measurement of the expression levels of the candidate genes^20,21^. Moreover, their applicability as a diagnostic tool in clinical practice has been extensively questioned in the literature mainly due to problems related to standardization, reproducibility and reliability^22–24^. In contrast, real-time PCR (qPCR) has been considered the “gold standard” in genetic expression studies^25^, and it has been demonstrated to offer a sustainable approach to validate microarrays discovery results with high accuracy, time savings and a relatively low cost^21,26,27^. In general, there is a good correlation between microarray and qPCR expression results^28^, but this correlation is strongly affected by technical as well as analytical factors^29–31^. Owing to the lack of experimental details in many qPCR-based assays, some studies have emphasized the need of high quality qPCR laboratory performance^32^.

A rapid bedside test device development based on these two transcripts is desirable but difficult to achieve due to technology limitations^33,34^. Meanwhile, a well optimized, cost- and time-effective qPCR-based protocol to discriminate between viral and bacterial infections could be easily implemented in the hospital molecular diagnostics laboratory routine.

As a first step for the translational application of this host expression signature, we developed a two-step RT-qPCR assay to test and validate the 2-transcript signature of genes *FAM89A* (RefSeq mRNA: NM_198552.2) and *IFI44L* (RefSeq mRNA: NM_006820.3). We tested the expression of these two genes in a new cohort of febrile children with confirmed bacterial infection, confirmed viral infection, and healthy controls. In contrast to previous studies that only considered bacterial and viral patients, we introduced a control group in order to assess the test performance in healthy subjects. Our results are discussed in the light of previous findings^15,16,19^.

## Results

### Description of the cohort

A total of 25 samples were collected from febrile patients with confirmed viral (*n* = 11) and bacterial (*n* = 14) infection in acute phase of the disease. In addition, we collected 10 samples from healthy controls for the comparisons.

All patients were of self-reported South-European ancestry. Demographic as well as some clinical parameters of the cohort are described in Table 1 and Supplementary Table S2. There were not statistically significant differences of demographic features and white blood cell types between bacterial and viral groups (Table 1).

**Table 1:** Demographic and clinical characteristics of the cohort.

Characteristics	All	Bacterial	Viral	Control	Bacterial vs. Viral (*P*-value)
n	35	14	11	10	—
Age; median (IQR)	2.2 (1.4–8.7)	2.3 (1.2–8.7)	3.5 (1.0–6.4)	1.6 (1.5–8.5)	0.809
Sex (% Female/Male)	(43/57)	(36/64)	(45/ 55)	(50/50)	0.697
Admitted to hospital (%)	84	93	72	—	—
PICU (%)	43	54	25	—	—
Admission time (days); median (IQR)	8.0 (6.0–13.0)	10.0 (10.0–15.0)	6.5 (5.5–7.0)	—	—
C-reactive protein (mg/L); median (IQR)	46.0 (19.0–241.0)	190.1 (71.25–198.8)	9.6 (4.2–43.5)	—	—
Neutrophils; median (IQR)	11.1 (7.5–14.6)	11.6 (7.6–14.8)	10.2 (6.2–13.3)	—	0.250
Lymphocytes; median (IQR)	1.7 (0.8–4.0)	1.4 (0.7–3.4)	2.4 (1.0–3.8)	—	0.654
Monocytes; median (IQR)	0.8 (0.4–1.2)	1.1 (0.6–2.0)	0.6 (0.3–1.0)	—	0.072
Days from symptoms (fever) to blood collection; mean	2.36	2.64	2.00	—	—
Maximum Tª before admission; median (IQR)	39.0 (38.2–39.5)	39.1 (38.6–39.9)	39.0 (38.1–39.1)	—	—
Pathogens (%)					
*Neisseria meningitidis*	—	42.9	—	—	—
*Streptococcus pneumoniae*	—	14.3	—	—	—
*Escherichia Coli*	—	21.4	—	—	—
*Staphylococcus aureus*	—	14.3	—	—	—
Group A *Streptococcus pyogenes*	—	7.1	—	—	—
Enterovirus	—	—	36.3	—	—
Respiratory syncytial virus	—	—	27.3	—	—
*Influenza* A	—	—	18.2	—	—
Adenovirus	—	—	18.2	—	—

The bacterial cohort consisted of six PICU admitted patients with sepsis and/or meningitis syndrome infected with *Neisseria meningitidis* (*n* = 5 meningococcus serogroup B and *n* = 1 meningococcus serogroup W135), three patients with focal pneumonia caused by *Streptococcus pneumoniae* (*n* = 2; one was a PICU patient) and GAS (*Group A Streptococcus pyogenes*; *n* = 1), three patients with *Escherichia coli* urinary tract infection, and two patients with bone infection and pyogenic infection with the detection of *Staphylococcus aureus* as causative pathogen.

The viral cohort included four patients with Enterovirus viral meningitis; three patients with bronchiolitis produced by respiratory syncytial virus (RSV), two adenovirus positive patients with respiratory illness (one PICU patient) and two patients with Influenza A virus infection (one admitted to PICU). All patients classified as “viral” were negative in the blood culture test result.

### Best reference gene selection

**Figure 1:**
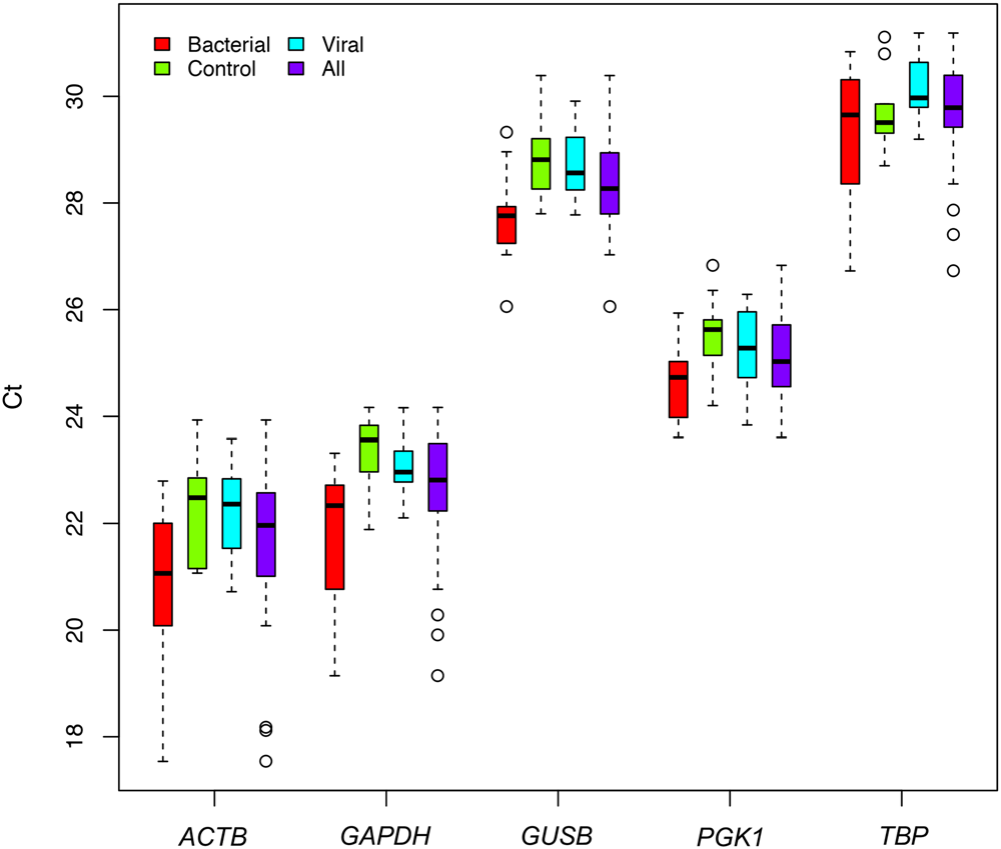
Boxplot of C_t_ raw data. Raw C_t_ data (y-axis) obtained from qPCR assay of candidate reference genes (x-axis) in viral, bacterial, and control cohorts. The box represents the interquartile range (25^th^ to the 75^th^) containing the middle 50% of the data, the line in the box represents the median, and the whiskers represent the ranges for the bottom 25% and the top 25% of the data values, excluding outliers.

The expression levels of a reference gene should be virtually constant under different conditions and tissues. The raw threshold cycle (C_t_; the PCR cycle at which a specific fluorescence becomes detectable) results obtained from each candidate (Supplementary Table S3) gene were represented in Fig. 1 and analyzed through descriptive statistics (mean, median, minimum, maximum and SD; Supplementary Table S4).

The mean C_t_ values of the candidate reference genes ranged from 21.6 to 29.7, indicating a moderate to relatively high expression of these genes in blood, with *ACTB* being the highest expressed gene and *TBP* the lowest. The highest gene expression stability between samples is represented by the reference genes *PGK1* (SD = 0.843), *GUSB* (SD = 0.907) and *TBP* (SD = 0.997), while *GAPDH* (SD = 1.200) and *ACTB* (SD = 1.483) showed the highest instability values. Instability of the latter two genes seems to derive from the cohort of bacterial patients (SD = 1.310 for *GAPDH*, and SD = 1.665 for *ACTB*).

**Figure 2:**
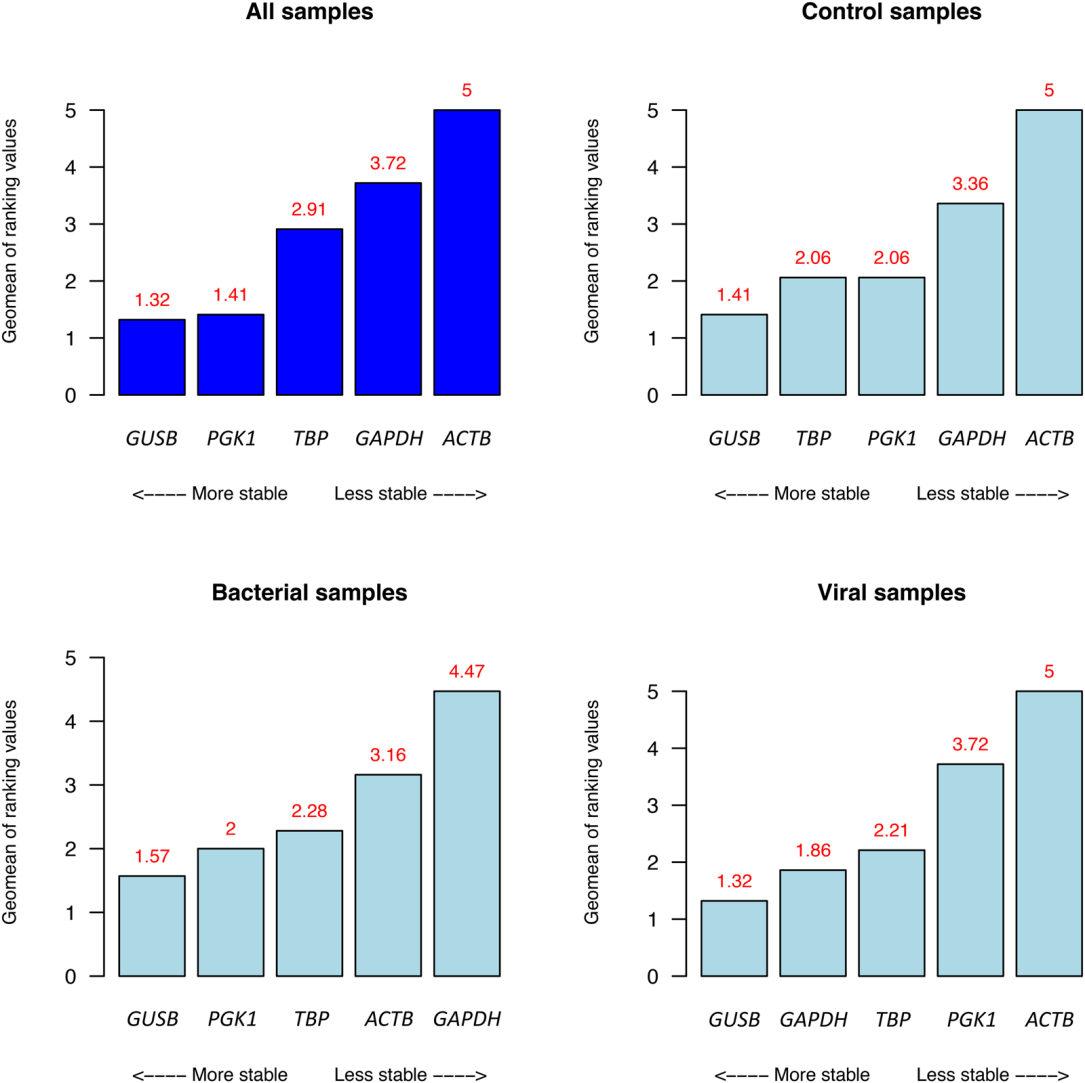
Comprehensive stability ranking results of the different candidate reference genes in the different cohorts.

Determination of stability using available algorithms for best reference gene selection provided similar results considering the whole cohort (Fig. 2). Results from comprehensive ranking (Supplementary Table S5), which uses the stability scores obtained from Bestkeeper, ∆C_t_ method, NormFinder and geNorm tools to calculate an overall ranking, showed that the most stable candidates (from high stability to less stability) are *GUSB* (1.32), *PGK1* (1.41) and *TBP* (2.91), while the genes with the worst stability values are *GAPDH* (3.72) and *ACTB* (5.00). This stability ranking is consistent when control, viral and bacterial patients are analyzed separately (with the exception of viral infected patients, in which *GAPDH* is ranked in second place, before *TBP* and *PGK1* genes), with *GUSB*, *PGK1* and *TBP* as the three most stable genes.

We additionally investigated the correlation between different algorithms by analyzing the correlation matrix obtained from the different stability rankings (Supplementary Fig. S1). The highest correlation was found to be between NormFinder *vs*. ∆C_t_ (Pearson r^2^ = 0.96; *P*-values range: 0.01–0.001) and RefFinder *vs*. ∆C_t_ (Pearson r^2^ = 0.96; *P*-values range: 0.01–0.001), whilst the lowest correlation was between NormFinder *vs*. Bestkeeper (Pearson r^2^ = 0.63; *P*-values range: 0.1–1). Despite the differences in stability rankings between the different methods, these results point to moderate to high correlation between them.

### Fold change calculation

We calculated the fold change of bacterial and viral patients through the delta-delta C_t_ method or 2^−∆∆Ct^ method^35^ using samples from healthy individuals as calibrator control (Supplementary Fig. S2). *FAM89A* gene yielded much lower expression values (mean C_t_ = 33.11) than *IFI44L* gene, and this was reflected in their fold change values with respect to control samples. Slight expression differences between bacterial and viral groups were recorded, with bacterial patients showing the highest *FAM89A* expression. Single gene normalization produced variable results, and reference genes with lower stability score (*GAPDH* and *ACTB*) yielded inverse expression pattern to *FAM89A* gene, pointing to problems derived from the use of only one gene to normalize the data, especially in genes with low expression values. This is also reflected in the correlation analysis using relative expression data (∆C_t_) of viral, bacterial and control groups (Supplementary Fig. S3). In general, despite the overall good correlation values between normalizations, the worst correlation (Pearson r^2^ < 0.5) was generated by gene pairs in which *GADPH* and *ACTB* were involved. This was noted when analyzing all samples together or the viral-bacterial group alone (all groups: *GAPDH*–*ACTB* [Pearson r^2^ = 0.41, *P*-value range: 0.05–0.01], *GAPDH*–*TBP* [Pearson r^2^ = 0.43, *P*-value range: 0.01–0.001]; viral-bacterial group: *GAPDH*–*ACTB* [Pearson r^2^ = 0.38, *P*-value range: 0.1–0.05], *GAPDH*–*TBP* [Pearson r^2^ = 0.37, *P*-value range: 0.1–0.05], *ACTB*–*PGK1* [Pearson r^2^ = 0.48, *P*-value range: 0.05–0.01]).

In addition, when we investigated the discrimination power of *FAM89A* using ∆C_t_ data we did not find consistent results among the different reference gene(s) used to normalize the expression (Supplementary Table S6). For the *FAM89A* gene, the use of multiple reference genes normalization produced more consistent results.

In the case of *IFI44L* gene, we recorded higher expression values (mean C_t_ = 27.67), and the fold change differences between viral and the other groups were clearly visible (Supplementary Fig. S2). Correlation between different normalization reference possibilities was very high in all cases (*P*-value = 0–0.001), including those pairs involving *GADPH* and *ACTB* (lower values: *ACTB*–*GAPDH*: Pearson r^2^ = 0.86; *GAPDH*–*TBP*: Pearson r^2^ = 0.85) (Supplementary Fig. S3).

Remarkably, the ability of *IFI44L* gene to separate viral group from bacterial and control groups is maintained constant regardless of the reference gene used to normalize the expression data (Supplementary Table S6; *post-hoc* analysis *P*-value range: 2.5 × 10^−4^–3.0 × 10^−6^ for viral *vs*. bacterial groups; *P*-values 8.0 × 10^−4^–3.6 × 10^−6^ for viral *vs*. control groups).

In both *FAM89A* and *IFI44L* genes we obtained very similar correlation results when control samples were removed from the analysis.

### Disease risk score stability

We investigated if DRS is influenced by the use of different references gene(s) (Supplementary Fig. S4).

We observed a very high correlation between DRS values independently of the reference gene(s) (Pearson r^2^ ranging from 0.97 to 1.00; *P*-values range: 0–0.001; Supplementary Fig. S5) in both the whole cohort and the viral-bacterial sub-cohort. In addition, we only observed statistically significant differences in DRS (Supplementary Table S7), calculated using the different reference gene options, between the following pairs: *ACTB vs*. *GUSB* (*P*-value = 0.042), *ACTB vs*. *TBP* (*P*-value = 0.007) and *GAPDH vs*. *TBP* (*P*-value = 0.016) considering all dataset; and only in one pair (*ACTB vs*. *TBP*; *P*-value = 0.044) when we removed control samples from the analysis. Genes considered less stable according to the stability ranking (*ACTB* and *GAPDH*) were involved in all significantly different DRS values.

When we compared the ability of the DRS to distinguish between viral and bacterial infections using different normalization references, we found a statistically significant difference in the DRS of children affected by bacterial compared to those with viral infections, in all cases and independently of the normalization option employed (Table 2; *P*-values range: 7.03 × 10^−5^–2.37 × 10^−4^). These differences were also evident when we examined the ROC curves obtained considering different normalization references (Supplementary Fig. S6; AUC: 89–93%).

**Table 2:** Statistical assessment of 2-transcript Disease Risk Score (DRS) assay to differentiate between viral and bacterial groups using different reference gene(s) to normalize the expression.

	RG	p-values			AUC (%)
		Shapiro-Wilk test	f-test (variances)	t-test (groups)	
Single RG normalization	*GUSB*	0.286	0.618	7.03 × 10^−5^	92.2
	*PGK1*	0.222	0.253	2.37 × 10^−4^	89.0
	*TBP*	0.288	0.927	8.62 × 10^−5^	93.5
	*GAPDH*	0.185	0.260	1.66 × 10^−4^	90.3
	*ACTB*	0.210	0.258	7.41 × 10^−5^	92.2
Multiple RG normalization	*GUSB-PGK1*	0.265	0.364	1.37 × 10^−4^	91.0
	*GUSB-PGK1-TBP*	0.296	0.495	1.04 × 10^−4^	92.2
	*GUSB-PGK1-TBP-GAPDH*	0.259	0.401	1.16 × 10^−4^	91.6

AUC: area under the ROC curve; RF: reference gene.

All these data point to a good overall stability of the DRS, hence the test result is not affected by the use of different references to normalize the expression data.

### Test performance

**Figure 3:**
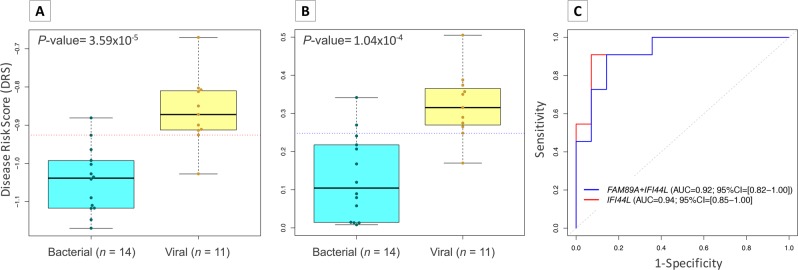
Evaluation of DRS test performance. (**A**) Boxplot of DRS values from bacterial and viral infected samples using 1-transcript signature (*IFI44L*). Horizontal red dotted line indicates the optimal threshold value. (**B**) Boxplot of DRS values from bacterial and viral samples using 2-transcript signature. Horizontal blue dotted line indicates the optimal threshold value. (**C**) ROC curves generated from 2-transcript (blue) and 1-transcript (red) (*IFI44L*) DRS with AUC values and 95% CI. We used a t-test to evaluate statistical differences in DRS values between bacterial and viral patients in panels (**A,B**). The box represents the interquartile range (25^th^ to the 75^th^) containing the middle 50% of the data, the line in the box represents the median, and the whiskers represent the ranges for the bottom 25% and the top 25% of the data values, excluding outliers.

When we analyzed DRS data obtained from the most stable three-gene combination (*GUSB*–*PGK1*–*TBP*) we observed that the expression signature can properly discriminate between viral and bacterial infections (Fig. 3A; *P*-value = 1.04 × 10^−4^; AUC = 92.2% [95% CI: 81.9–100%]). Overall, bacterial patients yielded lower DRS values (median = 0.104; IQR = [0.025–0.215]) than viral patients (median = 0.315; IQR = [0.270–0.365]). The optimal threshold point value representing the maximum difference between sensitivity and specificity (the point on the ROC curve farthest from the equality, i.e., from the diagonal) was 0.248 (Fig. 3A,C; sensitivity = 90.9% [95% CI: 72.7–100%]; specificity = 85.7% [95% CI: 64.3–100%]). The proportion of positive results that are true positives (PPV; in our case positive means bacterial infection) was 83.3% whereas the proportion of negative results that are true negatives (NPV; in our case negative means viral infection) was 92.3%. The high performance of the test fits very well with previous findings^15,16,19^.

DRS was not able to distinguish between bacterial and control samples (Supplementary Fig. S4). Despite this fact, control samples clearly show a narrower range of DRS values than the samples from bacterial infection, and they fall close to the upper DRS values of the bacterial patients.

Finally, we evaluated the ability of the assay to differentiate between the viral and bacterial group using only one of the two genes. Surprisingly, the results obtained using only expression data from *IFI44L* gene were similar to those derived from 2-gene DRS score (Fig. 3B,C; *P*-value = 3.59 × 10^−5^; AUC = 94.1% [95% CI: 85.3–100%]). In this case, the optimal threshold point value was −0.926, yielding sensitivity and specificity values of 90.9% and 92.8%, respectively (Fig. 3B).

**Figure 4:**
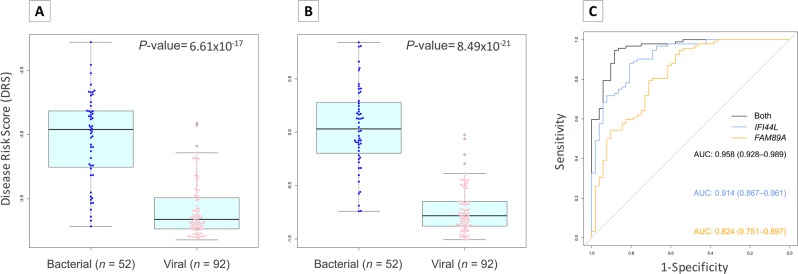
Evaluation of DRS test performance in microarray data from definitive bacterial and definitive viral infection in febrile children. (**A**) Boxplot of DRS values using 1-transcript signature (*IFI44L*). (**B**) Boxplot of DRS using 2-transcript signature. (**C**) ROC curves generated from 2-transcript (black) and 1-transcript ([*IFI44L*; blue], [*FAM89A*; yellow]) DRS with AUC values and 95% CI. We used a t-test to evaluate statistical differences in DRS values between bacterial and viral patients in panels (A,B). The box represents the interquartile range (25^th^ to the 75^th^) containing the middle 50% of the data, the line in the box represents the median, and the whiskers represent the ranges for the bottom 25% and the top 25% of the data values, excluding outliers.

To validate this unexpected result, we interrogated microarray data from Herberg *et al*.^16^ and similar values than those from the 2-transcripts were obtained for both definitively bacterial and viral samples (Fig. 4A, *P*-value = 6.61 × 10^−17^; Fig. 4B; *P*-value = 8.49 × 10^−21^; Fig. 4C; AUC = 0.91, 95% CI = [0.87–0.96]) and for probable bacterial and viral cohort (Supplementary Fig. S7A, *P*-value = 1.47 × 10^−7^; Supplementary Fig. S7B; *P*-value = 3.59 × 10^−5^; Supplementary Fig. S7C; AUC = 0.93, 95% CI = [0.85–1.00]).

**Figure 5:**
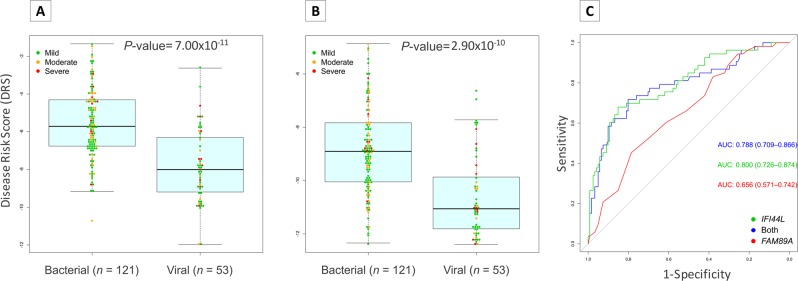
Evaluation of DRS test performance in whole transcript data from bacterial and viral diarrhea patients. (**A**) Boxplot of DRS values using 1-transcript signature (*IFI44L*). (**B**) Boxplot of DRS using 2-transcript signature. Dots indicate disease severity. Dots indicate disease severity. (**C**) ROC curves generated from 2-transcript (blue) and 1-transcript ([*IFI44L*; green], [*FAM89A*; red]) DRS with AUC values and 95% CI. We used a t-test to evaluate statistical differences in DRS values between bacterial and viral patients in panels (A,B). The box represents the interquartile range (25^th^ to the 75^th^) containing the middle 50% of the data, the line in the box represents the median, and the whiskers represent the ranges for the bottom 25% and the top 25% of the data values, excluding outliers.

Likewise, monogenic expression signature was tested in whole transcriptome data (RNA-seq) from patients suffering bacterial and viral diarrhea (Fig. 5). Consistently, we observed that *IFI44L* expression signal alone can seamlessly separate patients with diarrhea of bacterial and viral etiologies; the results being comparable to the DRS values obtained using both *FAM89A* and *IFI44L* together (Fig. 5A, *P*-value = 7.00 × 10^−11^; Fig. 5B; *P*-value = 2.90 × 10^−10^; Fig. 5C; AUC = 0.80, 95% CI = [0.73–0.87]).

Therefore, test performance is not compromised by the use of *IFI44L* expression alone.

## Discussion

Disease-associated biomarkers are currently a major research focus in the medical field, as their study and translational applications may contribute to understand, diagnose, prognosticate and treat patients in more efficient ways. Several biomarkers (proteins or mRNA) have been evaluated in the context of a broad range of infectious diseases^12,16,18,36^ and non-infectious complex diseases^37–40^ so far. Here, we have shown that a RT-qPCR assay for a 2-transcript host expression signature (*FAM89A* and *IFI44L* genes) inferred from microarray data is able to efficiently separate viral from bacterial infections. In addition, *IFI44L* expression signature alone seems to differentiate both groups, thus saving time, effort, and costs over the two genes signature. Blood-based host transcriptomics in pediatric patients constitute a promising tool for disease biomarker identification. A test based on host expression data offers additional benefits compared to the typical routine testing. Bacterial culture tests from sterile sites, routinely implemented in hospitals, usually cover the most common pathogens, often leading to false negative results, particularly when the infection resides in a non-accessible site, the patient was previously treated with antibiotics or if the causative pathogen is not interrogated^16,41,42^. Moreover, the time from admission to test results usually takes more than 24 hours. A rapid host-based expression assay could enhance the diagnosis of febrile children with viral or bacterial etiologies and will reduce the need of clinicians prescribing antibiotics as a preventive tool. Thus, an early differentiation between viral and bacterial patients will help improve triage in emergency departments, decrease the misuse of antibiotics, and guide the clinics to a more precise diagnosis. This will prevent the onset of antibiotics resistance as well as more broadly improving the quality of the health system.

As a first step towards establishing a host-based test in hospital routine, we have investigated two mRNA biomarkers, previously identified in microarray expression experiments^16^, in a custom relative expression RT-qPCR assay using commercially available Taqman probes. One of the most critical issues in relative expression experiments based on qPCR can be the selection of appropriate reference genes. It has been reported that the accuracy of the results obtained is strongly dependent on the selection of candidate reference genes. Validations of housekeeping genes must be specific of a particular experimental condition, and are a key component in assessing any new condition^32^. Because of this, important efforts have been devoted in the present study to the evaluation and selection of the most convenient reference genes. Our experiments indicate that the three most stable reference genes, from higher to lower stability, are *GUSB*, *PGK1* and *TBP* (Fig. 2). Discrepancies between stability rankings were previously reported and may be attributed to different algorithms implemented in the available methods^43^. Our data indicate that the well-known genes *GAPDH* and *ACTB* are too unstable; this could be due to their known variable expression patterns in different tissues and conditions^44,45^. In addition, it has been reported that the expression of these two genes can be modified during infection^46,47^. For routine applications, it is reasonable to strive for a balance between the benefits of adding a new reference gene to achieve extra precision during the normalization process, and the cost of including additional reference genes. Keeping this in mind and considering that the accepted recommendation is to use at least two reference genes to normalize qPCR data^48^, we chose to employ the three most stable reference candidates to normalize the qPCR expression of *FAM89A* and *IFI44L* genes.

We found that a RT-qPCR test based on DRS displays very good performance at distinguishing between viral and bacterial infections in febrile children (*P*-value = 1.04 × 10^−4^; AUC = 92.2% [95% CI: 81.9–100%]; Fig. 3A,C), with a potential clinical use for both “ruling in” (specificity >85%) and “ruling out” (sensitivity >90%) viral infections. These results are in good agreement with previous findings^15,16,19^. Contrary to microarrays and RNA-seq studies, DRS based on the RT-qPCR assay is higher in patients with viral infection and lower in patients affected by bacterial infection. This difference is due to the methodological differences between the microarray and qPCR techniques for measuring gene expression. Microarrays data work with expression intensity values (higher expression is related to higher intensity); hence, calculation of DRS from these data usually returns higher DRS values when *FAM89A* gene is highly expressed with respect to *IFI44L* gene, i.e., in bacterial samples. However, lower DRS values are expected in viral patients due to the higher expression of gene *IFI44L* compared to gene *FAM89A*. In contrast, qPCR uses C_t_ values and, therefore, higher expression is represented by lower C_t_ values. When the DRS formula is applied to qPCR data, high expression of gene *IFI44L* and gene *FAM89A* results in low ∆C_t(*IFI44L*)_ and ∆C_t(*FAM89A*)_, respectively. Hence, samples with higher expression of gene *IFI44L* and lower expression of gene *FAM89A* (viral patients) will give a higher DRS value than samples with lower expression of gene *IFI44L* and higher expression of gene *FAM89A* (bacterial samples). Notwithstanding this difference between DRS scales obtained from microarray-based and qPCR-based studies, this does not interfere with the test performance.

The DRS 2-transcript test was not able to differentiate between control and bacterial patients but it clearly distinguished the viral group from bacterial and control groups. Note, however, that in the clinical practice the utility of this test lies in its ability to efficiently separate viral from bacterial children with fever; in such contexts, controls do not necessarily play a role.

The expression signals produced by *FAM89A* gene showed low expression level, low expression differences between viral and bacterial cohort and high variability when different normalization controls are employed. In contrast, *IFI44L* gene produces a more robust and reliable expression signal and it is less prone to variability derived from the use of different normalization references. Surprisingly, our results indicate that a test based on a single expression signal generated by the *IFI44L* gene not only suffices to differentiate accurately between viral and bacterial children with fever, but it also performs slightly better than the 2-transcript signature (Fig. 3B,C; *P*-value = 3.59 × 10^−5^; AUC = 94.1% [95% CI: 85.3–100%]; sensitivity >90% and specificity >92%). In addition, we succeeded in validating this finding in two independent cohorts composed by microarray data from febrile children with definitively bacterial and viral infection^16^ (Fig. 4A,C; *P*-value = 6.61 × 10^−17^; AUC = 0.91, 95% CI = [0.87–0.96]) and whole transcriptome data from acute diarrhea of viral and bacterial aetiology (Fig. 5A,C; *P*-value = 7.00 × 10^−11^; AUC = 0.80; 95% CI: 0.73–0.87). Both microarray and RNA-seq data also allowed to confirm the limited informativeness of the *FAM89A* signature. This simplification of the test to a single gene expression provides the opportunity to save time, effort and costs.

Some interesting issues remain to be investigated with regards to this transcriptomic signature, namely: (*i*) the evolution of gene expression over time from the onset of fever, and (*ii*) variations in its expression patterns with regards to the causative pathogen, co-infections and/or mixed viral/bacterial infections, (*iii*) non-infectious febrile illness (i.e. rheumatoid arthritis) and (*iv*) adult patients.

The present study represents an important step towards the implementation of a functional host transcriptomic-based test capable of distinguishing between viral and bacterial infections in febrile children, thus contributing to a more accurate diagnosis and treatment of pediatric patients.

## Methods

### Sample size and statistical power calculation

In order to estimate the minimum sample size required to detect differences between viral and bacterial groups with high probability (0.8–0.9) we performed a *post-hoc* statistical power analysis based on these two transcripts data (*IFI44L* and *FAM89A*) previously published by Herberg *et al*.^16^ retrieved from GEO database (Gene Expression Omnibus; https://www.ncbi.nlm.nih.gov/geo/; accession number: GSE72829). We calculated the Disease Risk Score (DRS; see Data analysis in M&M section for more details about DRS calculation) mean from definitively bacterial (*n* = 52) and definitively viral (*n* = 92) samples to obtain delta parameter (mean difference) of each group as well as standard deviation (SD) from both groups together. We used pwr R package^49^ to carry out the analysis with a significance level of 0.05 and a statistical power of 80% and 90%.

This analysis indicates that a sample size of *n* = 8.4 samples per group is needed to discriminate between viral and bacterial group with a statistical power of 80% (delta = 0.69; SD = 0.47). When we raised the power to 90% we obtained a sample size of *n* = 10.8 samples per group. Therefore, we need a minimum of 8 samples per group to have enough statistical power to detect true differences between both groups.

### Sample collection

The study was approved by the Ethical Committee of Clinical Investigation of Galicia (CEIC ref. 2016/331) and was conducted according to the principles of the Declaration of Helsinki and in accordance with all applicable Spanish normative, namely, Law for Biomedical Research (Law 14/2007-3 of July), Law 41/2002 of Autonomy of the Patient, Decree SAS/3470/2009 for Observational Studies and Law 15/1999 of Data Protection. Written informed consent was obtained from a parent or legal guardian for each subject before study inclusion.

PAXgene^TM^ tubes were used to collect 2.5 ml blood samples from 25 febrile children admitted to Hospital Clínico Universitario from Santiago de Compostela (Spain) with confirmed bacterial (*n* = 14) and confirmed viral infections (*n* = 11). The clinical selection and classification of the patients was carried out according to the decision scheme previously published by Herberg *et al*.^16^. Additionally, we collected healthy control samples (*n* = 10) for the comparisons. Demographic and clinical data from these patients and controls are summarized in Table 1.

PAXgene^TM^ RNA samples were collected within 24 hours of admission to hospital following the manufacturer guidelines, stored at −20 °C for 24 hours and then stored at −80 °C until the RNA extraction.

### RNA extraction and quantification

Samples were thawed and left at room temperature overnight before extraction in order to increase the RNA yields. RNA extraction was performed using PAXgene Boold RNA kit (Qiagen). Samples were treated with RNase-free DNase I during the extraction process to destroy all DNA present in the sample that could interfere in downstream applications. After extraction, RNA amount and purity of the samples was measured using NanoDrop 1000 (ThermoFisher) (Supplementary Table S1).

### RT-qPCR reactions

RT-PCR reaction was carried out to obtain cDNA from RNA samples. Some preliminary assays were performed in order to check and select an adequate RNA and cDNA input concentration and to test the expression level of the genes in our samples. High level of C_t_ variation is indicative of poor precision and consequently low fold changes are more difficult to quantify accurately. Therefore, we normalized RNA input for RT-PCR reaction to 100 ng/μl using treated ultrapure DEPC-H_2_O. A volume of 10 μl of this dilution was used in the reaction (total RNA template was 1 μg). Firstly, 1 μl of Random primers p(dN)6 (Roche) and 1 μl of DNTPs mix (Invitrogen) were added to the template. Random primers are hexamers of random sequences that hybridize to random sites to perform conversion of the whole RNA to cDNA. The mixture was then heated to 65 °C for 5 minutes and immediately placed in ice. After a brief centrifugation, 4 μl of 5× First-Strand Buffer, 2 μl of DTT (0.1 M) and 1 μl of Recombinant RNasin® Ribonuclease Inhibitor (Promega) were added and the mixture was incubated at 25 °C for 2 minutes. Finally, 1 μl (200 units) of SuperScript™ II Reverse Transcriptase (Invitrogen) was introduced to the mix for a final reaction volume of 20 μl. The RT-PCR reaction was carried out under the following conditions: 15 minutes at 25 °C, 50 minutes at 42 °C, 15 minutes at 70 °C.

For the qPCR assay we used 10 μl of Master mix (2×) Kapa Probe Fast qPCR Kit, 7 μl of MiliQ water, 1 μl of the TaqMan assay mix and 2 μl of the cDNA (1:5 dilution). In order to test the assay precision, three replicates of each gene per sample were run. Replicates with standard deviations over 0.5 were repeated. qPCR reaction was carried out in a StepOnePlus Real-Time PCR System (Applied Biosystems) with the following parameters: 2 minutes at 50 °C, 10 minutes at 95 °C, 40 cycles of 15 seconds at 95 °C, and 1 minute at 60 °C.

### Selection of best reference gene candidates

The selection of the best reference gene(s) for the assay was conducted through testing five of the most commonly used human reference genes: *PGK1* (Phosphoglycerate Kinase 1), *ACTB* (beta-actin), *GUSB* (beta-glucuronidase), *TBP* (TATA-binding protein) and *GAPDH* (glyceraldehyde 3-phosphate dehydrogenase) (TaqMan probes IDs: Hs00943178_g1, Hs99999903_m1, Hs00984230_m1, Hs00939627_m1, Hs00427621_m1, Hs03929097_g1, respectively) (Table 3). We selected best reference gene candidates using different software packages available: geNorm^50^, NormFinder^51^, BestKeeper^52^, and the ∆C_t_ method^53^. BestKeeper and ∆C_t_ method use raw Ct data for calculations, while geNorm and NormFinder work on relative quantities. Finally, we calculated the overall final ranking using the web-based tool RefFinder^54^, which considers the ranking of the above-mentioned tools to assign a final weight to each candidate gene, and calculates the geometric mean of their weights to build the final ranking.

**Table 3:** List of candidate reference genes and genes of interest evaluated in the present study. RG: reference gene; GOI: gene of interest. Chromosome locations are referred to the reference genome GRCh38.

RG/GOI	Symbol	Protein name	Accession number	Chromosome location	Taqman probe
RG	*ACTB*	β-actin	NM_001101	Chr.7: 5527148–5530601	Hs99999903_m1
RG	*GAPDH*	Glyceraldehyde 3-phosphate dehydrogenase	NM_002046	Chr.12: 6534405–6538375	Hs03929097_g1
RG	*GUSB*	β-glucuronidase	NM_000181	Chr.7: 65960684–65982314	Hs00939627_m1
RT	*PGK1*	Phosphoglycerate kinase 1	NM_000291	Chr.X: 78104169–78126827	Hs00943178_g1
RG	*TBP*	TATA box-binding protein	NM_003194	Chr.6: 170554333–170572870	Hs00427621_m1
GOI	*IFI44L*	Interferon induced protein 44 like	NM_006820	Chr.1: 78620382–78646145	Hs00915294_g1
GOI	*FAM89A*	Family with sequence similarity 89 member A	NM_198552	Chr.1: 231018958–231040249	Hs00293357_m1

We constructed the correlation matrix between rankings using R software^55^.

### Gene expression of *FAM89A* and *IFI44L*

We followed a qPCR TaqMan-based approach to test the host expression balance between these two transcripts in febrile pediatric patients. Probe IDs corresponding to *FAM89A* and *IFI44L* transcripts were first retrieved from Chip GeneChip^TM^ HG-U133 Plus 2.0 (Affymetrix Inc; 226448_at and 204439_at, respectively). TaqMan^TM^ (ThermoFisher Scientific) probes exactly matching the array probes from GeneChip^TM^ were then selected (assay IDs: Hs00293357_m1 for the *FAM89A* transcript and Hs00915294_g1 for the transcript *IFI44L*) in order to avoid potential variations due to the use of different probe sequences^29^.

### Data analysis

A relative quantification method 2^−∆∆Ct^ ^35^ was used to normalize the samples with respect to reference genes and obtain fold change values of viral and bacterial cohorts using control samples as calibrator. TaqMan probes are extensively checked, and therefore an efficiency close to 100% can be assumed (see details: https://assets.thermofisher.com/TFS-Assets/LSG/Application-Notes/cms_040377.pdf; last access 29/01/2019).

We evaluated the statistical significance in demographics and cell types between viral and bacterial cohorts using one-factor ANOVA or Wilcoxon test for numeric variables and depending on the data (normality, equality of variances and homoscedasticity), and Fisher’s exact test for categorical variables.

Different statistical tests were carried out on relative expression data from *FAM89A* and *IFI44L* genes depending on the assumptions of normality (Shapiro-Wilk) and homoscedasticity (Breusch-Pagan): one factor ANOVA, robust one-way ANOVA and Kruskal-Wallis. Post-hoc tests analyses were performed using Tukey test and Games-Howell test, as appropriate.

We followed Kaforou *et al*.^56^ to compute the Disease Risk Score (DRS) for each sample. We first obtained individual DRS values using normalized values (∆C_t_). We then incremented the scale of the ∆C_t_ values by a factor of 10 in order to avoid negative values when the log-scale is applied. The final DRS formula was as follows:

$$DR{S}_{sample}=(log[{{\rm{\Delta }}\mathrm{Ct}}_{FAM89}+10])-(log[{{\rm{\Delta }}\mathrm{Ct}}_{IFI44L}+10])$$

After DRS calculation we studied the impact of using different single and multiple reference genes on DRS stability. Firstly, we performed a correlation analysis to check the extent of correlation between DRS calculated from different reference gene combinations. Secondly, we investigated if there were significant differences in DRS using different reference genes as normalizers. Due to the fact that the data did not meet the normal distribution (Shapiro-Wilk test), we used a non-parametric Kruskal-Wallis and a post-hoc Games-Howell tests to carry out the analysis. Finally, we used a t-test for independent samples to evaluate the statistical significance of differences in DRS values between bacterial *vs*. viral patients and how the use of different reference gene to normalize the data affects the capacity of DRS to differentiate between these two groups. The latter was done after testing that the data followed a normal distribution (Shapiro-Wilk test) and met the assumption of equality of variances (f-test).

We investigated the discrimination power of the individual *IFI44L* gene signature using the formula:

$$DR{S}_{sample}=-\,(log[{{\rm{\Delta }}\mathrm{Ct}}_{IFI44L}+10])$$

We maintained the negative sign to avoid points of the ROC (Receiver Operating Characteristic) curve being located below the diagonal, meaning an inverse relationship between the measure and the event probability (i.e., the larger the measure, the less likely the event).

We used the ROCR package^57^ to evaluate the predictive accuracy of the 2-transcript signature, by creating a ROC curve to asses test performance; this represents the sensitivity (true positive rate) *vs*. 1 − specificity (false positive rate) for all possible values of the cut-point between two status. In addition, we calculated the area under the ROC curve (AUC) and the confidence interval as well as the positive predictive value (PPV) and negative predictive value (NPV) to measure the diagnostic potential of the expression signature. The optimal cut-point value, defined as the point on the ROC curve that classifies most of the individuals correctly (maximizing sensitivity and specificity), was calculated using the Youden method included in the OptimalCutPoints R package^58^. The calculation of the confidence intervals for sensitivity and the specificity was based on a stratified bootstrap resampling.

Graphical representations and statistical tests were carried out using R software^55^.

### Monogenic signature validation in microarray and whole transcriptome data

We evaluated the discrimination power of the 1-transcript signature (*IFI44L*) in microarray data from Herberg *et al*.^16^ retrieved from GEO database (accession number: GSE72829) consisting of *n* = 52 definitively bacterial and *n* = 92 definitively viral samples from febrile children. Furthermore, we also checked the performance in probable viral and probable bacterial cohorts included from the same study (n = 42 probable bacterial; *n* = 5 probable viral). Samples were processed and analyzed using same methodology described in^16^. Normalization of raw data was carried using the same method as in^16^ (*lumi* R package^59^).

We also tested the *IFI44L* signature in whole transcriptome data (RNA-Seq) previously analyzed in Barral-Arca *et al*.^19^ and also available in GEO database (accession number: GSE69529). RNA expression dataset includes Mexican children (*n* = 174) younger than 10 years of age with acute diarrhea associated with a single viral or bacterial pathogen [dataset]^60^. Processing of RNA-seq data was carried out as described previously^19^. Normalization of the raw whole transcriptome data was performed through the same procedure used in^19^ (DESeq2R package^61^).

Graphical representations and statistical tests were carried out using R software^55^.

## Supplementary information

Supplementary material
