# Supplementary material for: A qPCR expression assay of IFI44L gene differentiates viral from bacterial infections in febrile children
Source: Sci Rep. 2019 Aug 13;9:11780. doi: 10.1038/s41598-019-48162-9 (PMC6692396; doi:10.1038/s41598-019-48162-9)
Supplement: Supplementary file 1 — Supplementary material [file 41598_2019_48162_MOESM1_ESM.pdf]

A qPCR expression assay of *IFI44L* gene differentiates viral from bacterial infections in febrile children

Alberto Gómez-Carballa, Miriam Cebey-López, Jacobo Pardo-Seco, Ruth Barral-Arca, Irene Rivero-Calle, Sara Pischedda, María José Currás-Tuala, José Gómez-Rial, Francisco Barros, Federico Martinón-Torres, Antonio Salas

Supplementary Figures

Supplementary Figure S1. Correlation matrix comparing the different methods to best candidate reference gene selection according to gene stability.

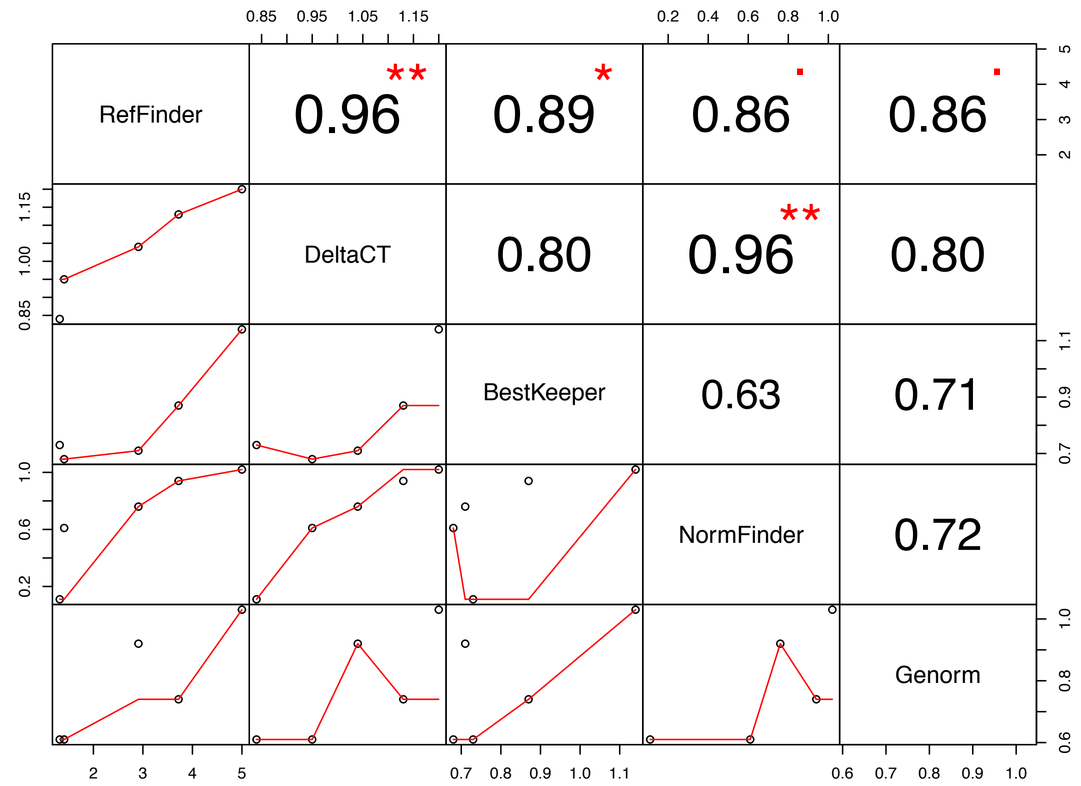

Supplementary Figure S2. Barplots representing *FAM89A* and *IFI44L* fold change values in viral and bacterial groups using control group as calibrator and different normalization references.

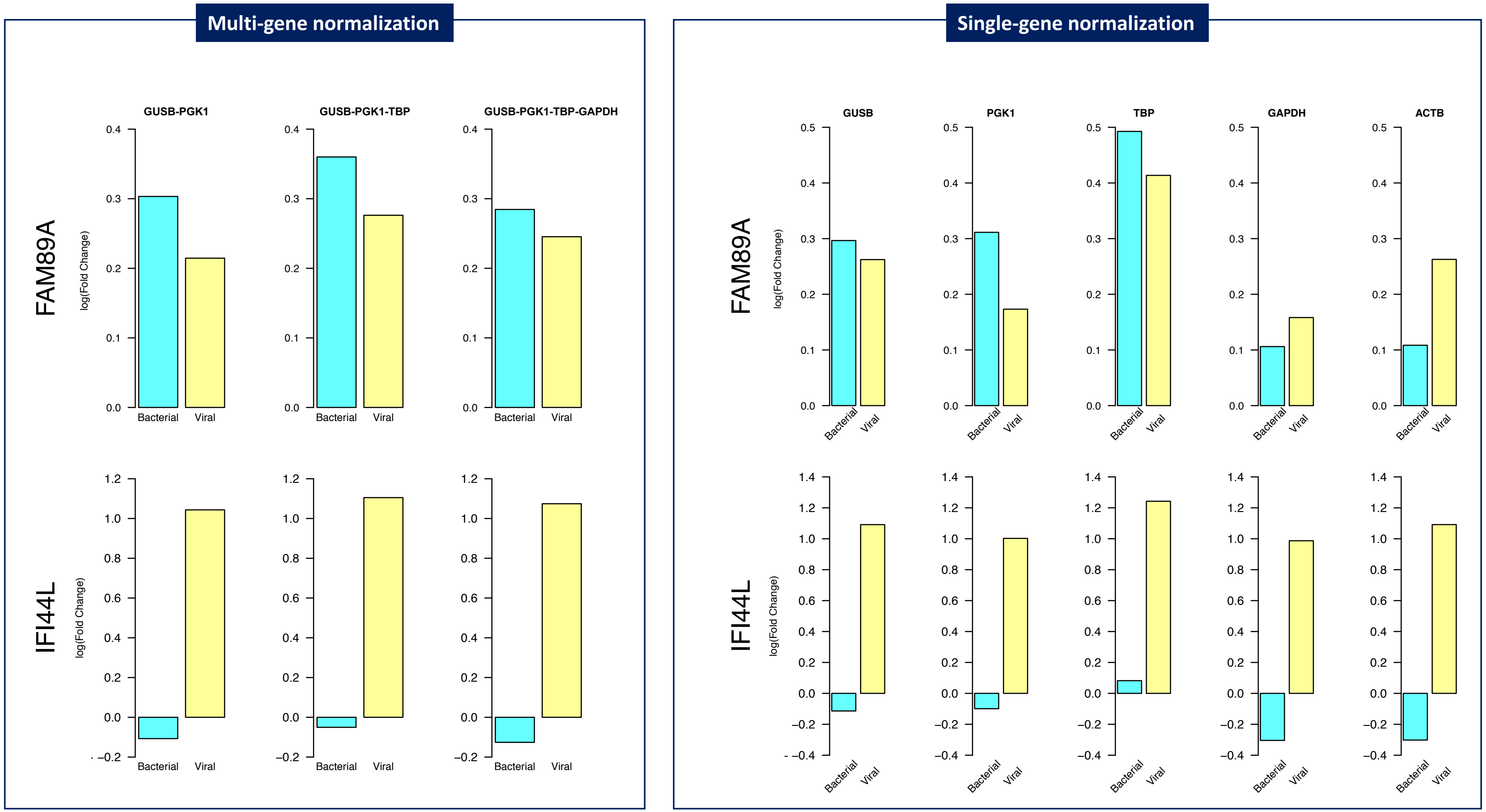

Supplementary Figure S3. Correlation matrixes of *FAM89A* and *IFI44L* relative expression data comparing different normalization references. Letters located in the diagonal indicate different reference gene options: A (*GUSB*), B (*GUSB–PGK1*), C (*GUSB–PGK1–TBP*), D (*GUSB–PGK1–TBP–GAPDH*), E (*PGK1*), F (*TBP*), G (*GAPDH*), H (*ACTB*).

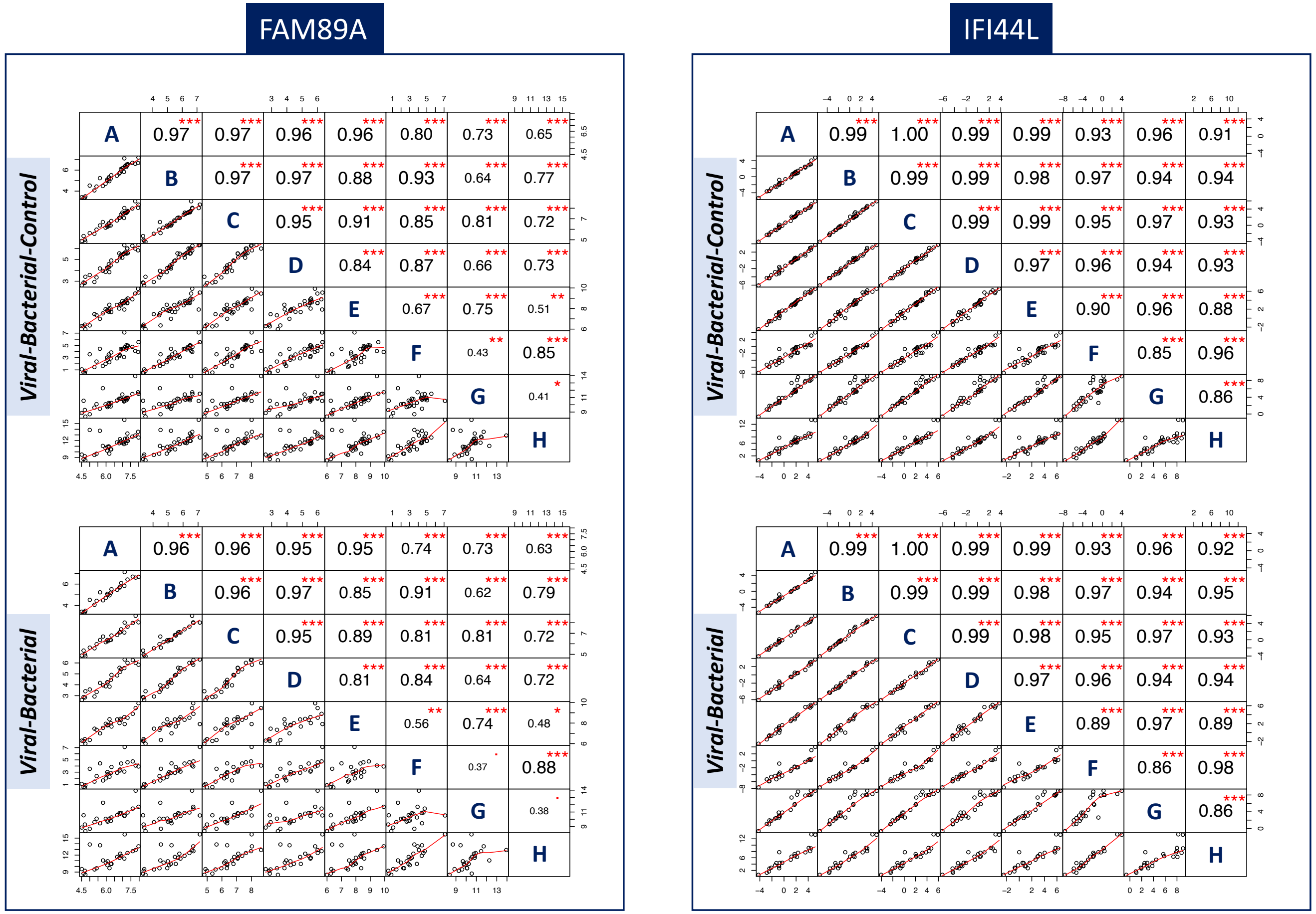

Supplementary Figure S4. Boxplots of 2-transcipt DRS values from bacterial, viral and control groups using different reference genes to normalize the expression.

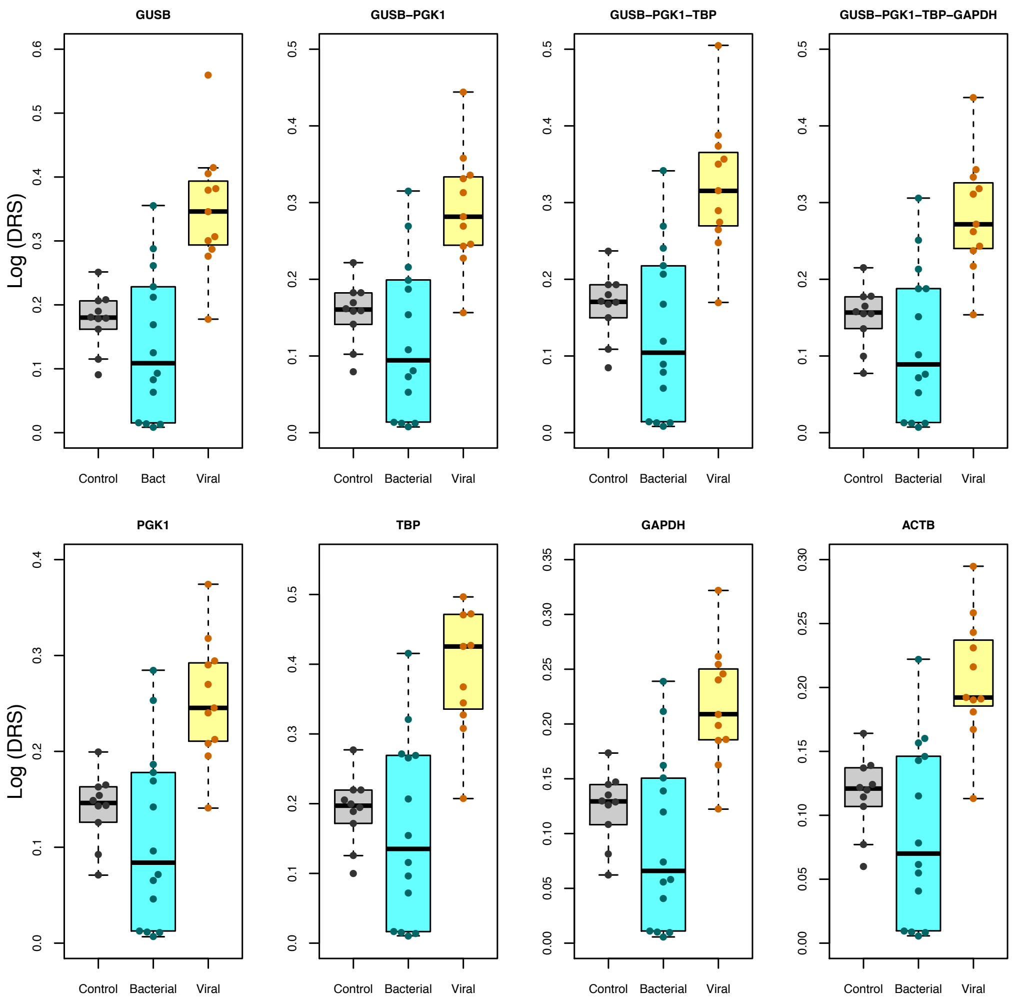

Supplementary Figure S5. Correlation matrices of 2-transcript DRS comparing different normalization references (diagonal).

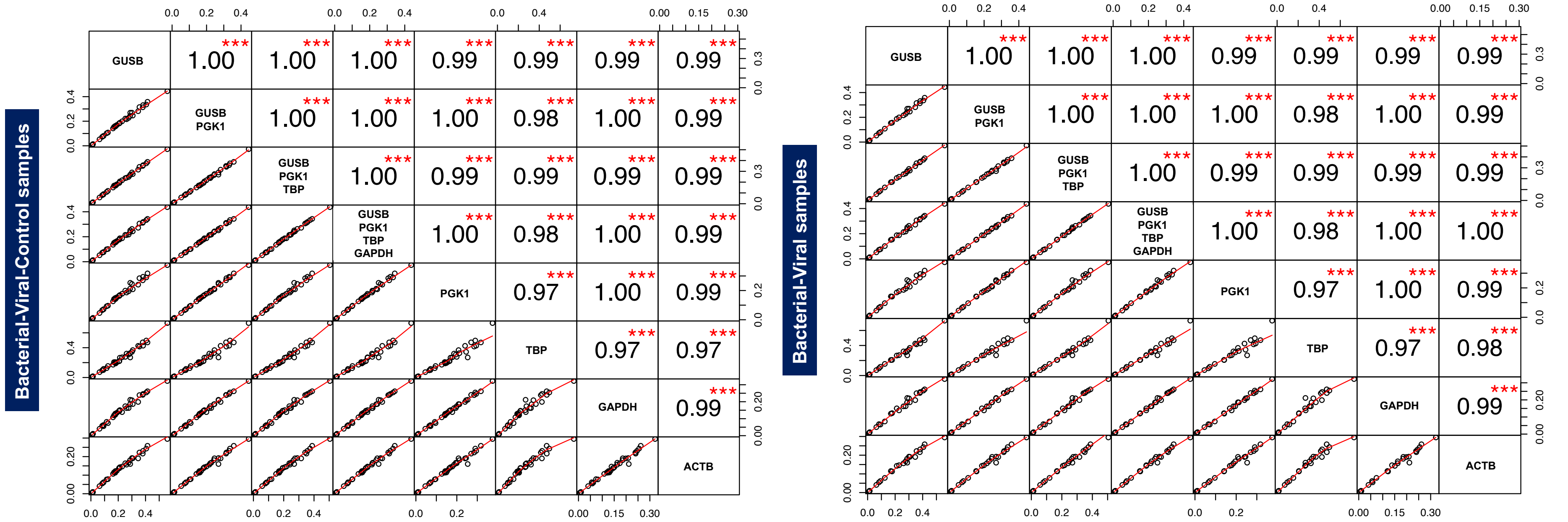

Supplementary Figure S6. ROC curves of DRS calculated from different reference gene(s) combinations. (A) normalized expression data from a single reference gene and (B) normalized expression data from multiple reference genes, to study 2-trancript DRS test stability. AUC values are also represented.

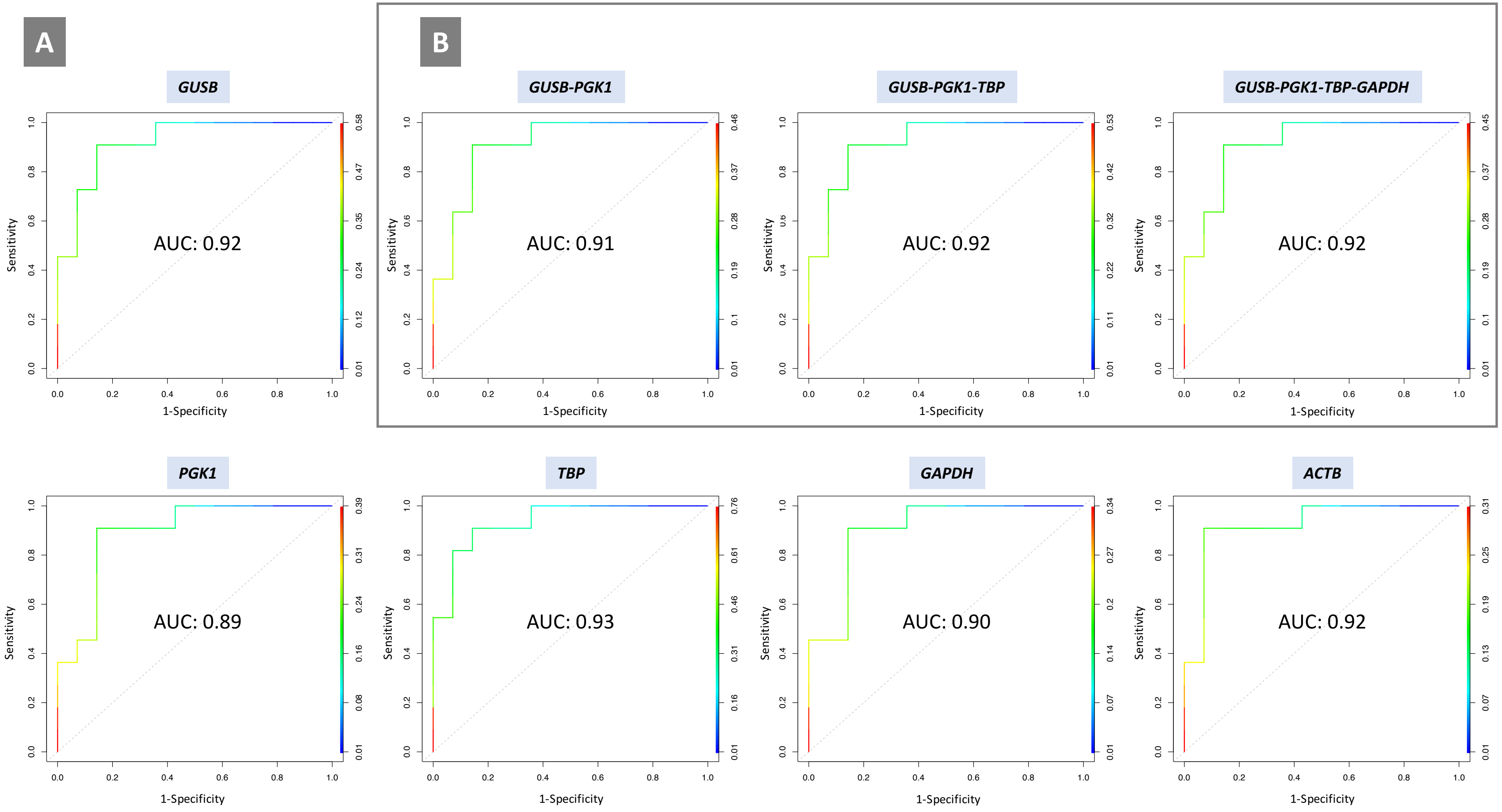

Supplementary Figure S7. Evaluation of DRS test performance in microarray data from probable bacterial and probable viral infection in febrile children. (A) Boxplot of DRS values using 1-transcript signature (IFI44L). (B) Boxplot of DRS using 2-transcript signature. C) ROC curves generated from 2-transcript (black) and 1-transcript ([IFI44L; blue], [FAM89A; yellow]) DRS with AUC values and 95% CI. We used a t-test to evaluate the statistical significance of differences in DRS values between bacterial and viral patients in panels (A) and (B) (*P*-value is noted). The box represents the interquartile range (25th to the 75th) containing the middle 50% of the data, the line in the box represents the median and the whiskers represent the ranges for the bottom 25% and the top 25% of the data values, excluding outliers.

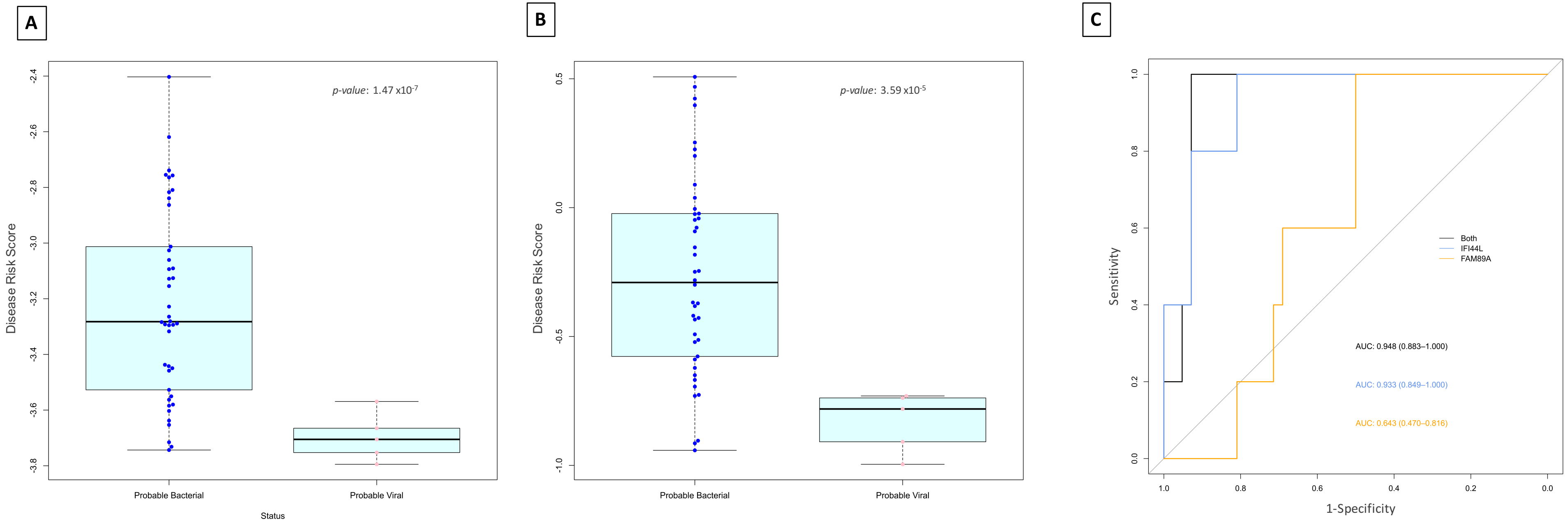

Supplementary Tables

Supplementary Table S1. Quality (A260/280) and quantity (ng/µl) measurements of the RNA samples.

Cohort	Sample ID	ng/ul	A260/280
Bacterial	B1	107	2.03
Bacterial	B10	251.8	1.98
Bacterial	B11	111.9	2.03
Bacterial	B12	167.8	2.01
Bacterial	B13	83.6	2.05
Bacterial	B14	238.9	2.08
Bacterial	B2	109	2.03
Bacterial	B3	269.8	2.07
Bacterial	B4	207.5	2.07
Bacterial	B5	147.8	1.9
Bacterial	B6	252.4	2.07
Bacterial	B7	283.3	2.06
Bacterial	B8	137.5	2.05
Bacterial	B9	108.8	2.05
Control	C11	261.1	2.06
Control	C12	175.3	2.07
Control	C13	255.5	2.07
Control	C14	175.1	2.07
Control	C15	215.9	2.06
Control	C16	222	2.05
Control	C2	106.3	2.06
Control	C3	99.7	2.03
Control	C4	100.1	2.07
Control	C6	98.2	2.02
Viral	V1	181	2.05
Viral	V10	114.2	2.04
Viral	V13	200	1.9
Viral	V14	80.9	1.9
Viral	V2	136.1	2.08
Viral	V3	69	1.9
Viral	V5	140.5	2.06
Viral	V6	251.4	1.9
Viral	V7	286.4	2.07
Viral	V8	141.8	1.9
Viral	V9	103.6	2.07

Supplementary Table S2. Detailed description of the clinical syndrome, causative pathogen and pathogen detection site of bacterial and viral patients.

Sample ID	Clinical syndrome	Detection site	Detection method	Pathogen that matches with the syndrome	Categorization
**B1**	Focal pneumonia+Empyema	Blood	Culture	Streptococcus pneumoniae	**Definite bacterial infection**
**B10**	Sepsis/Meningitis	Blood/CSF	Culture/PCR	Neisseria meningitidis W135	**Definite bacterial infection**
**B11**	Focal pneumonia+pleural effusion	Pleural liquid	Culture	Group A Streptococcus pyogenes	**Definite bacterial infection**
**B12**	Sepsis/Meningitis	Blood/CSF	Culture/PCR	Neisseria meningitidis B	**Definite bacterial infection**
**B13**	Sepsis/Meningitis	CSF	PCR	Neisseria meningitidis B	**Definite bacterial infection**
**B14**	Sepsis	Blood	Culture	Neisseria meningitidis B	**Definite bacterial infection**
**B2**	Focal pyogenic infection	Blood	Culture	Staphylococcus aureus	**Definite bacterial infection**
**B3**	Pyelonehritis	Blood/Urine	Culture	Escherichia coli	**Definite bacterial infection**
**B4**	Sepsis/Meningitis	CSF	PCR	Neisseria meningitidis B	**Definite bacterial infection**
**B5**	Sepsis	Blood	Culture	Neisseria meningitidis B	**Definite bacterial infection**
**B6**	Meningitis /Focal pneumonia	CSF	Culture	Streptococcus pneumoniae	**Definite bacterial infection**
**B7**	Urinary tract infection	Urine	Culture	Escherichia coli	**Definite bacterial infection**
**B8**	Urinary tract infection	Urine	Culture	Escherichia coli	**Definite bacterial infection**
**B9**	Bone infection	Articular aspirate	Culture	Staphylococcus aureus	**Definite bacterial infection**
**V1**	Febrile illness/Respiratory illness	Nasopharyngeal swab	PCR	Adenovirus	**Definite viral infection**
**V10**	Febrile illness/Respiratory illness	Nasopharyngeal aspirate	Direct immunofluorescence	Adenovirus	**Definite viral infection**
**V13**	Bronchiolithis	Nasopharyngeal aspirate	Direct immunofluorescence/PCR	Respiratory syncytial virus	**Definite viral infection**
**V14**	Flu-like illness	Nasopharyngeal aspirate	Direct immunofluorescence	Influenza A	**Definite viral infection**
**V2**	Meningitis	CFS	PCR	Enterovirus	**Definite viral infection**
**V3**	Meningitis	CFS	PCR	Enterovirus	**Definite viral infection**
**V5**	Meningitis	CFS	PCR	Enterovirus	**Definite viral infection**
**V6**	Bronchiolithis	Nasopharyngeal aspirate	Direct immunofluorescence	Respiratory syncytial virus	**Definite viral infection**
**V7**	Bronchiolithis	Nasopharyngeal aspirate	Direct immunofluorescence	Respiratory syncytial virus	**Definite viral infection**
**V8**	Fever/viriasis	Nasopharyngeal aspirate	Direct immunofluorescence/Immunochromatography	Influenza A	**Definite viral infection**
**V9**	Meningitis	CFS	PCR	Enterovirus	**Definite viral infection**

Supplementary Table S3. Raw C_t results obtained from qPCR analysis (SD: standard deviation; C_t: threshold cycle)

Patient group	Sample Name	Target Name	Cт Mean	Cт SD
Bacterial	B1	ACTB	22.10749944	0.211292444
Bacterial	B10	ACTB	22.79511451	0.248041958
Bacterial	B11	ACTB	20.08116340	0.316745102
Bacterial	B12	ACTB	18.17631721	0.14690046
Bacterial	B13	ACTB	17.53716087	0.073547766
Bacterial	B14	ACTB	18.11574745	0.203547359
Bacterial	B2	ACTB	20.95109367	0.031697132
Bacterial	B3	ACTB	20.36261177	0.028297555
Bacterial	B4	ACTB	20.83031654	0.023548299
Bacterial	B5	ACTB	22.14953423	0.110614948
Bacterial	B6	ACTB	22.14077759	0.346594363
Bacterial	B7	ACTB	21.48986244	0.455580652
Bacterial	B8	ACTB	21.17649078	0.28970018
Bacterial	B9	ACTB	21.71056366	0.093387976
Control	C11	ACTB	22.85270119	0.117699616
Control	C12	ACTB	22.50065422	0.071276776
Control	C13	ACTB	23.93084145	0.041297328
Control	C14	ACTB	22.56804085	0.055717461
Control	C15	ACTB	22.71550083	0.273120637
Control	C16	ACTB	23.5781002	0.367071807
Control	C2	ACTB	21.06855774	0.093600802
Control	C3	ACTB	21.96365166	0.015830193
Control	C4	ACTB	21.15398598	0.078396499
Control	C6	ACTB	21.11449051	0.018531127
Viral	V1	ACTB	22.51643372	0.191271171
Viral	V10	ACTB	22.21755981	0.125931144
Viral	V13	ACTB	22.36164284	0.036983039
Viral	V14	ACTB	23.58377075	0.113426946
Viral	V2	ACTB	20.85639381	0.16352357
Viral	V3	ACTB	22.57996368	0.147466764
Viral	V5	ACTB	20.72017097	0.215403453
Viral	V6	ACTB	21.43855095	0.060992103
Viral	V7	ACTB	23.35408211	0.062957235
Viral	V8	ACTB	22.9373951	0.061539851
Viral	V9	ACTB	22.73521996	0.067996338
Bacterial	B1	FAM89A	30.83327357	0.102168771
Bacterial	B10	FAM89A	32.2567215	0.030375402
Bacterial	B11	FAM89A	31.60918617	0.174855992
Bacterial	B12	FAM89A	33.83978653	0.095375732
Bacterial	B13	FAM89A	31.41231728	0.065162808
Bacterial	B14	FAM89A	31.84594727	0.030053496
Bacterial	B2	FAM89A	33.84226608	0.097805463
Bacterial	B3	FAM89A	31.41438293	0.029801084
Bacterial	B4	FAM89A	30.04650879	0.019356117
Bacterial	B5	FAM89A	32.81961823	0.116712213
Bacterial	B6	FAM89A	30.65001488	0.230723515
Bacterial	B7	FAM89A	34.18722534	0.295884222
Bacterial	B8	FAM89A	32.55935287	0.178552583
Bacterial	B9	FAM89A	33.38117218	0.123354726
Control	C11	FAM89A	34.78633881	0.068546094
Control	C12	FAM89A	34.45547867	0.158003792
Control	C13	FAM89A	35.55267334	0.145061702
Control	C14	FAM89A	35.10797119	0.224798471
Control	C15	FAM89A	34.376252	0.374618295
Control	C16	FAM89A	35.12350082	0.32709828
Control	C2	FAM89A	34.30958176	0.191271171
Control	C3	FAM89A	32.82543945	0.052022405
Control	C4	FAM89A	32.12509537	0.157612443
Control	C6	FAM89A	33.43808746	0.034944795
Viral	V1	FAM89A	32.20562871	0.251244147
Viral	V10	FAM89A	32.66957092	0.087773509
Viral	V13	FAM89A	31.8833313	0.014990726
Viral	V14	FAM89A	33.25386047	0.116368458
Viral	V2	FAM89A	34.40213013	0.436654359
Viral	V3	FAM89A	32.80054474	0.080193654
Viral	V5	FAM89A	32.96580505	0.171559826

Viral	V6	FAM89A	33.79992294	0.066244923
Viral	V7	FAM89A	33.79601669	0.137881771
Viral	V8	FAM89A	33.83935547	0.201009855
Viral	V9	FAM89A	34.60550308	0.204357103
Bacterial	B1	GAPDH	20.28407288	0.087177216
Bacterial	B10	GAPDH	22.64853477	0.018191254
Bacterial	B11	GAPDH	20.7711544	0.16264987
Bacterial	B12	GAPDH	23.31471062	0.258448869
Bacterial	B13	GAPDH	22.71408081	0.101686135
Bacterial	B14	GAPDH	22.39276695	0.074698351
Bacterial	B2	GAPDH	19.90884781	0.083359458
Bacterial	B3	GAPDH	19.14368248	0.158102557
Bacterial	B4	GAPDH	20.76605797	0.174273968
Bacterial	B5	GAPDH	22.7543087	0.012599407
Bacterial	B6	GAPDH	22.27603149	0.088016175
Bacterial	B7	GAPDH	22.71754456	0.428576171
Bacterial	B8	GAPDH	22.10373878	0.362450063
Bacterial	B9	GAPDH	22.81356812	0.20931676
Control	C11	GAPDH	23.82407188	0.113771744
Control	C12	GAPDH	23.83096504	0.123389997
Control	C13	GAPDH	24.14214134	0.191271171
Control	C14	GAPDH	23.59804916	0.143562287
Control	C15	GAPDH	23.67652893	0.262886256
Control	C16	GAPDH	24.1657486	0.100275777
Control	C2	GAPDH	23.46746826	0.064174406
Control	C3	GAPDH	22.96660805	0.09281417
Control	C4	GAPDH	21.88668251	0.031083016
Control	C6	GAPDH	22.67123032	0.001095144
Viral	V1	GAPDH	22.10421753	0.087279715
Viral	V10	GAPDH	22.89881706	0.064061433
Viral	V13	GAPDH	23.01795959	0.033813544
Viral	V14	GAPDH	23.16841698	0.02584647
Viral	V2	GAPDH	22.68534851	0.151585743
Viral	V3	GAPDH	22.96286774	0.002584108
Viral	V5	GAPDH	22.19153786	0.075155392
Viral	V6	GAPDH	22.86863136	0.11710085
Viral	V7	GAPDH	23.54419518	0.04492243
Viral	V8	GAPDH	24.16075897	0.046996716
Viral	V9	GAPDH	23.74048615	0.127928883
Bacterial	B1	GUSB	27.95595614	0.042075012
Bacterial	B10	GUSB	29.3277092	0.089723557
Bacterial	B11	GUSB	26.05183411	0.337472707
Bacterial	B12	GUSB	27.52637672	0.099549443
Bacterial	B13	GUSB	27.84195518	0.092341922
Bacterial	B14	GUSB	27.67903709	0.088418022
Bacterial	B2	GUSB	27.84035683	0.056144677
Bacterial	B3	GUSB	27.46257782	0.133208826
Bacterial	B4	GUSB	27.20935059	0.157975733
Bacterial	B5	GUSB	28.9597168	0.098725356
Bacterial	B6	GUSB	27.24297523	0.348738939
Bacterial	B7	GUSB	27.92786598	0.267349273
Bacterial	B8	GUSB	27.02847099	0.191271171
Bacterial	B9	GUSB	28.52396393	0.154535294
Control	C11	GUSB	29.20523071	0.10078121
Control	C12	GUSB	28.92201996	0.017761895
Control	C13	GUSB	30.38960266	0.07305485
Control	C14	GUSB	28.77670288	0.005788617
Control	C15	GUSB	28.86887169	0.058020268
Control	C16	GUSB	29.50861168	0.118678167
Control	C2	GUSB	28.30209923	0.044578947
Control	C3	GUSB	28.2623539	0.312240809
Control	C4	GUSB	27.84618568	0.051973596
Control	C6	GUSB	27.79780197	0.149666488
Viral	V1	GUSB	28.37133535	0.187239622
Viral	V10	GUSB	28.21881104	0.088585667
Viral	V13	GUSB	29.27719688	0.066123128
Viral	V14	GUSB	29.90982056	0.032755855
Viral	V2	GUSB	28.56390381	0.28584528
Viral	V3	GUSB	28.55895805	0.030925671
Viral	V5	GUSB	27.77684975	0.376625627
Viral	V6	GUSB	27.79217529	0.130265981
Viral	V7	GUSB	28.8916378	0.084914707

Viral	V8	GUSB	29.61716461	0.03743412
Viral	V9	GUSB	29.18592262	0.104822643
Bacterial	B1	IFI44L	28.3586216	0.104959231
Bacterial	B10	IFI44L	30.50461197	0.160670534
Bacterial	B11	IFI44L	26.59154701	0.221114695
Bacterial	B12	IFI44L	25.9318676	0.047944076
Bacterial	B13	IFI44L	30.94119263	0.049615122
Bacterial	B14	IFI44L	31.39826393	0.091213346
Bacterial	B2	IFI44L	27.29953003	0.0470405
Bacterial	B3	IFI44L	27.93076324	0.034473166
Bacterial	B4	IFI44L	29.79635811	0.191271171
Bacterial	B5	IFI44L	26.55922127	0.058854785
Bacterial	B6	IFI44L	30.24409485	0.234459862
Bacterial	B7	IFI44L	25.10074043	0.305403829
Bacterial	B8	IFI44L	26.56575584	0.170235366
Bacterial	B9	IFI44L	30.80992889	0.21757485
Control	C11	IFI44L	28.84585571	0.029855922
Control	C12	IFI44L	29.16104698	0.143142655
Control	C13	IFI44L	30.83475685	0.028235059
Control	C14	IFI44L	28.93391609	0.0574384
Control	C15	IFI44L	27.55944633	0.36336077
Control	C16	IFI44L	29.84741974	0.136686996
Control	C2	IFI44L	28.92222023	0.027944246
Control	C3	IFI44L	29.43231964	0.151187733
Control	C4	IFI44L	29.4243145	0.074447475
Control	C6	IFI44L	27.88807297	0.121295258
Viral	V1	IFI44L	25.2016449	0.254463106
Viral	V10	IFI44L	23.90198708	0.106612153
Viral	V13	IFI44L	24.95925331	0.029730543
Viral	V14	IFI44L	26.97307968	0.008673484
Viral	V2	IFI44L	25.17340279	0.132509068
Viral	V3	IFI44L	25.91637039	0.027731951
Viral	V5	IFI44L	27.86662483	0.14246051
Viral	V6	IFI44L	25.80592346	0.057350427
Viral	V7	IFI44L	24.63241577	0.047481652
Viral	V8	IFI44L	23.53855133	0.078432761
Viral	V9	IFI44L	25.5846405	0.102096438
Bacterial	B1	PGK1	24.57128588	0.085367545
Bacterial	B10	PGK1	24.80212021	0.055063341
Bacterial	B11	PGK1	23.60890198	0.307620198
Bacterial	B12	PGK1	25.93564415	0.073992707
Bacterial	B13	PGK1	25.05731773	0.191271171
Bacterial	B14	PGK1	24.84452057	0.02959205
Bacterial	B2	PGK1	24.38299751	0.041967079
Bacterial	B3	PGK1	23.88458443	0.079495907
Bacterial	B4	PGK1	23.61092949	0.010789379
Bacterial	B5	PGK1	24.8897686	0.101490937
Bacterial	B6	PGK1	24.6574192	0.280075401
Bacterial	B7	PGK1	25.28822136	0.382258683
Bacterial	B8	PGK1	23.97909546	0.080619946
Bacterial	B9	PGK1	25.02650452	0.224795774
Control	C11	PGK1	25.80862236	0.138073891
Control	C12	PGK1	25.58380127	0.028963339
Control	C13	PGK1	26.83051872	0.056962684
Control	C14	PGK1	25.55394363	0.085007161
Control	C15	PGK1	25.85899099	0.333969727
Control	C16	PGK1	26.36032677	0.118107475
Control	C2	PGK1	25.75952721	0.043821406
Control	C3	PGK1	25.14301109	0.19091098
Control	C4	PGK1	24.20117188	0.176068842
Control	C6	PGK1	24.83243179	0.187241256
Viral	V1	PGK1	23.83847809	0.1900924
Viral	V10	PGK1	24.67484983	0.381337563
Viral	V13	PGK1	25.58842659	0.036146171
Viral	V14	PGK1	25.90872955	0.074518323
Viral	V2	PGK1	24.47222328	0.184422001
Viral	V3	PGK1	25.02218246	0.05807903
Viral	V5	PGK1	24.55700874	0.203741223
Viral	V6	PGK1	25.27843666	0.038417961
Viral	V7	PGK1	26.13157082	0.10645552
Viral	V8	PGK1	26.00800133	0.187945053
Viral	V9	PGK1	26.28646088	0.040554035

Bacterial	B1	TBP	30.26392746	0.191271171
Bacterial	B10	TBP	30.80063248	0.050619375
Bacterial	B11	TBP	28.35820961	0.37391603
Bacterial	B12	TBP	26.72585869	0.262974143
Bacterial	B13	TBP	27.86510468	0.117132843
Bacterial	B14	TBP	27.40779686	0.165918112
Bacterial	B2	TBP	29.76553917	0.184796542
Bacterial	B3	TBP	29.77502251	0.169930115
Bacterial	B4	TBP	29.53719139	0.136273906
Bacterial	B5	TBP	30.83573151	0.138732061
Bacterial	B6	TBP	29.78657532	0.467372864
Bacterial	B7	TBP	29.43255615	0.435049623
Bacterial	B8	TBP	29.46862793	0.49012664
Bacterial	B9	TBP	30.4935112	0.201186806
Control	C11	TBP	29.81539917	0.118451454
Control	C12	TBP	29.43815613	0.143108547
Control	C13	TBP	31.10685158	0.169756532
Control	C14	TBP	29.57775307	0.232283756
Control	C15	TBP	29.92817434	0.172708751
Control	C16	TBP	30.78953552	0.061754767
Control	C2	TBP	29.41057396	0.030723667
Control	C3	TBP	29.30977821	0.252083719
Control	C4	TBP	28.96174622	0.010970379
Control	C6	TBP	28.69813919	0.06923008
Viral	V1	TBP	29.94618416	0.04370073
Viral	V10	TBP	29.79943275	0.231584787
Viral	V13	TBP	30.7964077	0.120825306
Viral	V14	TBP	30.89377594	0.120132677
Viral	V2	TBP	30.47776222	0.113915756
Viral	V3	TBP	29.78895187	0.042385567
Viral	V5	TBP	29.53377724	0.108878255
Viral	V6	TBP	29.19589424	0.191271171
Viral	V7	TBP	29.9506855	0.043601394
Viral	V8	TBP	31.18650246	0.010755503
Viral	V9	TBP	30.19477272	0.100608915

Supplementary Table S4. Descriptive statistical analysis of the candidate reference genes.

Reference Gene	Group	Max	Min	Median	Mean	Standard Deviation
PGK1	All	26.83051872	23.60890198	25.02434349	25.05621683	0.842901198
	Bacterial	25.93564415	23.60890198	24.72976971	24.60995079	0.664538661
	Viral	26.28646088	23.78578091	25.15030956	25.12934576	0.874110689
	Control	26.83051872	24.20117188	25.67166424	25.59323457	0.744234691
GUSB	All	30.38960266	26.05183411	28.28222656	28.33408202	0.906817281
	Bacterial	29.3277092	26.05183411	27.75969696	27.7555819	0.819626621
	Viral	29.90982056	27.40555	28.56143093	28.63077715	0.77361538
	Control	30.38960266	27.79780197	28.82278728	28.78794804	0.793237575
TBP	All	31.18650246	26.72585869	29.78079891	29.67086218	0.997325346
	Bacterial	30.83573151	26.72585869	29.65136528	29.32259178	1.267668169
	Viral	31.18650246	28.83449936	29.94843483	30.04988718	0.69997761
	Control	31.10685158	28.69813919	29.5079546	29.70361074	0.752762311
GAPDH	All	24.1657486	19.14368248	22.78393841	22.60554187	1.200781349
	Bacterial	23.31471062	19.14368248	22.33439922	21.75779288	1.31070111
	Viral	24.16075897	21.61767578	22.9308424	22.91340939	0.715114659
	Control	24.1657486	21.88668251	23.63728905	23.42294941	0.716996289
ACTB	All	23.93084145	17.53716087	22.03557555	21.64838702	1.48302391
	Bacterial	22.79511452	17.53716087	21.06379223	20.68744669	1.665145299
	Viral	23.58377075	20.72017097	22.43903828	22.18926287	0.973953667
	Control	23.93084145	21.06855774	22.53434753	22.34465246	1.011700122

Supplementary Table S5. Stability values of candidate reference genes calculated through different methods available. (SD: standard deviation).

		Comprehensive Ranking	Method			
			**Delta CT**	**BestKeeper**	**NormFinder**	**Genorm**
	Reference Genes	*Rank (Geomean of ranking values)*	*Rank (Average of SD)*	*Rank (Average of SD)*	*Rank (Stability value)*	*Rank (Average expression Stability value)*
**All**	*GUSB*	1 (1.32)	1 (0.84)	3 (0.73)	1 (0.11)	1 (0.61)
	PGK1	2 (1.41)	2 (0.95)	1 (0.68)	2 (0.61)	1 (0.61)
	TBP	3 (2.91)	3 (1.04)	2 (0.71)	3 (0.76)	3 (0.92)
	ACTB	5 (5.00)	5 (1.20)	5 (1.14)	5 (1.02)	4 (1.03)
	GAPDH	4 (3.72)	4 (1.13)	4 (0.87)	4 (0.94)	2 (0.74)
**Viral+Bacterial**	*GUSB*	1 (1.32)	1 (0.84)	3 (0.73)	1 (0.11)	1 (0.61)
	PGK1	2 (1.41)	2 (0.95)	1 (0.63)	2 (0.61)	1 (0.61)
	TBP	3 (2.91)	3 (1.04)	2 (0.71)	3 (0.76)	3 (0.92)
	ACTB	5 (5.00)	5 (1.20)	5 (1.14)	5 (1.02)	4 (1.03)
	GAPDH	4 (3.72)	4 (1.13)	4 (0.86)	4 (0.94)	2 (0.74)
**Control**	*GUSB*	1 (1.41)	1 (0.34)	4 (0.59)	1 (0.12)	1 (0.25)
	PGK1	3 (2.06)	2 (0.36)	1 (0.53)	3 (0.23)	2 (0.29)
	TBP	2 (2.06)	3 (0.37)	3 (0.57)	2 (0.22)	1 (0.25)
	ACTB	5 (5.00)	5 (0.49)	5 (0.82)	5 (0.43)	4 (0.40)
	GAPDH	4 (3.36)	4 (0.44)	2 (0.55)	4 (0.36)	3 (0.34)
**Bacterial**	*GUSB*	1 (1.57)	1 (1.16)	2 (0.58)	1 (0.20)	2 (1.11)
	PGK1	2 (2.00)	2 (1.30)	1 (0.51)	2 (0.82)	3 (1.27)
	TBP	3 (2.28)	3 (1.35)	3 (0.99)	3 (0.94)	1 (0.70)
	ACTB	4 (3.16)	4 (1.61)	5 (1.31)	5 (1.42)	1 (0.70)
	GADPH	5 (4.47)	5 (1.62)	4 (1.13)	4 (1.41)	4 (1.41)
**Viral**	*GUSB*	1 (1.32)	1 (0.52)	3 (0.58)	1 (0.18)	1 (0.31)
	PGK1	4 (3.72)	4 (0.64)	4 (0.67)	3 (0.48)	3 (0.53)
	TBP	3 (2.21)	3 (0.63)	2 (0.50)	4 (0.50)	1 (0.31)
	ACTB	5 (5.00)	5 (0.75)	5 (0.72)	5 (0.64)	4 (0.62)
	GAPDH	2 (1.86)	2 (0.56)	1 (0.45)	2 (0.32)	2 (0.48)

Supplementary Table S6. **Statistical differences between bacterial, viral and control groups using relative expression data from *FAM89A* and *IFI44L* genes and different reference genes**. Cells in red colour indicate no statistical differences between the groups mentioned (p-value > 0.05).

Genes of Interest	Reference gene/s	Statistical tests p-values					Post-hoc analysis p-values		
		Normality test	Homoscedasticityis test	Parametric & Homoscedasticityis	Parametric & Non-homoscedasticityis	Non-parametric	Homoscedasticityis	Non-homoscedasticityis/normality	Groups
		Shapiro-Wilk	*Breusch-Pagan*	*One factor ANOVA*	*Robust one way ANOVA*	*Kruskal-Wallis*	*Tukey*	*Games-Howell*	
**IFI44L**	***ACTB***	0.010	0.016	-		1.5E-4	-	0.460	Bacterial vs. Control
								1.6E-4	Bacterial vs. Viral
								7.2E-5	Control vs. Viral
	GUSB	0.639	0.053	1.7E-6		-	0.840	-	Bacterial vs. Control
							3.0E-6		Bacterial vs. Viral
							5.3E-5		Control vs. Viral
	PGK1	0.664	0.048		2.3E-5		-	0.880	Bacterial vs. Control
								2.5E-4	Bacterial vs. Viral
								5.3E-5	Control vs. Viral
	TBP	0.130	0.015		1.2E-6		-	0.920	Bacterial vs. Control
								2.4E-4	Bacterial vs. Viral
								3.6E-6	Control vs. Viral
	GAPDH	0.231	0.051	8.3E-6			0.400	-	Bacterial vs. Control
							6.8E-6		Bacterial vs. Viral
							8.8E-4		Control vs. Viral
	GUSB-PGK1	0.865	0.050	5.4E-6			0.87	-	Bacterial vs. Control
							8.9E-6		Bacterial vs. Viral
							1.3E-4		Control vs. Viral
	GUSB-PGK1-TBP	0.937	0.042		4.1E-6		-	0.960	Bacterial vs. Control
								8.4E-5	Bacterial vs. Viral
								1.1E-5	Control vs. Viral
	GUSB-PGK1-TBP-GAPDH	0.805	0.054	2.9E-6			0.820	-	Bacterial vs. Control
							4.7E-6		Bacterial vs. Viral
							9.0E-5		Control vs. Viral
**FAM89A**	***ACTB***	0.731	0.015	-	0.194		-	0.830	Bacterial vs. Control
								0.740	Bacterial vs. Viral
								0.170	Control vs. Viral
	GUSB	0.717	0.136	0.075		-	0.079	-	Bacterial vs. Control
							0.962		Bacterial vs. Viral
							0.161		Control vs. Viral
	PGK1	0.458	0.161	0.028			0.022	-	Bacterial vs. Control
							0.413		Bacterial vs. Viral
							0.310		Control vs. Viral
	TBP	0.165	0.011		0.002		-	0.021	Bacterial vs. Control
								0.897	Bacterial vs. Viral
								0.006	Control vs. Viral
	GAPDH	0.031	0.006			0.177	-	0.680	Bacterial vs. Control
								0.920	Bacterial vs. Viral
								0.180	Control vs. Viral
	GUSB-PGK1	0.771	0.152	0.041			0.033	-	Bacterial vs. Control
							0.711		Bacterial vs. Viral
							0.198		Control vs. Viral
	GUSB-PGK1-TBP	0.882	0.097	0.022			0.019	-	Bacterial vs. Control
							0.772		Bacterial vs. Viral
							0.107		Control vs. Viral
	GUSB-PGK1-TBP-GAPDH	0.813	0.087	0.053			0.054	-	Bacterial vs. Control
							0.937		Bacterial vs. Viral
							0.133		Control vs. Viral

Supplementary Table S7. Statistical differences in DRS calculated from the different normalization references.

	Test	All samples	Viral-Bacterial samples
		p-value	*p-value*
	Kruskal-Wallis	0.001	0.011
	POST-HOC Games-Howell		
**Gene/s used to normalize the expression data**	*ACTB vs. GAPDH*	0.999	1.000
	ACTB vs. GUSB	0.042	0.179
	ACTB vs. GUSB-PGK1	0.298	0.578
	ACTB vs. GUSB-PGK1-TBP	0.106	0.310
	ACTB vs. GUSB-PGK1-TBP-GAPDH	0.407	0.675
	ACTB vs. PGK1	0.798	0.926
	ACTB vs. TBP	0.007	0.044
	GAPDH vs. GUSB	0.097	0.287
	GAPDH vs. GUSB-PGK1	0.526	0.756
	GAPDH vs. GUSB-PGK1-TBP	0.227	0.467
	GAPDH vs. GUSB-PGK1-TBP-GAPDH	0.655	0.838
	GAPDH vs. PGK1	0.951	0.984
	GAPDH vs. TBP	0.017	0.073
	GUSB vs. GUSB-PGK1	0.964	0.987
	GUSB vs. GUSB-PGK1-TBP	0.999	1.000
	GUSB vs. GUSB-PGK1-TBP-GAPDH	0.916	0.968
	GUSB vs. PGK1	0.570	0.776
	GUSB vs. TBP	0.976	0.985
	GUSB-PGK1 vs. GUSB-PGK1-TBP	0.999	1.000
	GUSB-PGK1 vs. GUSB-PGK1-TBP-GAPDH	1.000	1.000
	GUSB-PGK1 vs. PGK1	0.988	0.996
	GUSB-PGK1 vs. TBP	0.514	0.673
	GUSB-PGK1-TBP vs. GUSB-PGK1-TBP-GAPDH	0.994	0.998
	GUSB-PGK1-TBP vs. PGK1	0.837	0.930
	GUSB-PGK1-TBP vs. TBP	0.842	0.908
	GUSB-PGK1-TBP-GAPDH vs. PGK1	0.998	0.999
	GUSB-PGK1-TBP-GAPDH vs. TBP	0.406	0.585
	PGK1 vs. TBP	0.142	0.300
